# Synergistic Passivation of Bulk and Heterojunction Defects via Dual‐Functioned Interlayer Treatment for High‐Efficiency Sb_2_(S,Se)_3_ Solar Cells

**DOI:** 10.1002/advs.202515777

**Published:** 2025-10-05

**Authors:** Muhammad Ishaq, Yiming Zhong, Muhammad Abbas, Adil Mansoor, Boyang Fu, Jun Zhao, Zhenghua Su, Shuo Chen, Rajwali Khan, Zhuanghao Zheng, Guangxing Liang

**Affiliations:** ^1^ Shenzhen Key Laboratory of Advanced Thin Films and Applications Key Laboratory of Optoelectronic Devices and Systems of the Ministry of Education and Guangdong Province State Key Laboratory of Radio Frequency Heterogeneous Integration College of Physics and Optoelectronic Engineering Shenzhen University Shenzhen Guangdong 518060 China; ^2^ National Water and Energy Center United Arab Emirates University Al Ain 15551 United Arab Emirates

**Keywords:** antisite defects, heterojunction engineering, iodide thermal diffusion, lattice mismatching, spike‐like CBO, vaccany defects

## Abstract

The performance of Sb_2_(S,Se)_3_‐based photovoltaics is largely limited by intrinsic defects in the absorber layer and suboptimal electronic characteristics at the heterojunction. Extensive efforts have been devoted to improving the quality of the absorber layer and distinctly modifying the electron transport layer (ETL) through targeted doping and surface treatments. Herein, a unified approach is presented that simultaneously addresses both of these challenges and establishes a paradigm shift from conventional sequential optimization strategies. A thin layer of KI is spin‐coated between the CdS and Sb_2_(S,Se)_3_, whereupon annealing, iodide ions diffuse into the Sb_2_(S,Se)_3_ film, promoting grain growth, enhancing crystallization, and elevating the work function. Simultaneously, the KI treatment enhances the conductivity of the CdS, adjusts its energy band positions, and creates a favorable spike‐like alignment at the heterojunction, effectively suppressing interfacial carrier recombination. Furthermore, the KI treatment also mitigates detrimental vacancy defects (V_S/Se_) and reduces antisite defects (Sb_S/Se_) within Sb_2_(S,Se)_3_ film. Consequently, the champion device exhibits a remarkable power conversion efficiency (PCE) of 10.06%, a significant improvement over the control device's PCE of 8.14%. This work presents a holistic approach to optimizing both absorber quality and ETL characteristics, offering a promising pathway to enhance the performance of Sb_2_(S,Se)_3_‐based solar cells.

## Graphical abstract

This work introduces a dual‐function KI treatment that simultaneously enhances Sb_2_(S,Se)_3_ absorber quality and CdS heterojunction properties. Iodide diffusion passivates bulk defects while improving crystallization and modifies the ETL's band alignment. The resulting spike‐like interface suppresses recombination, achieving a 10.06% PCE. The unified approach demonstrates how single‐step processing can address multiple limitations in antimony chalcogenide photovoltaics.

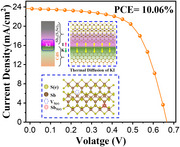

## 1 Introduction

Metal chalcogenides have garnered significant attention for their photovoltaic (PV) application over the past decade, with cadmium telluride and copper indium gallium selenide standing out as the key examples. Solar cells based on both these materials have demonstrated power conversion efficiencies (PCEs) over 22%.^[^ ^1^ ^]^ However, the toxic nature of cadmium (Cd) and the limited availability of the indium (In) and gallium (Ga) pose a challenge to their commercial applications.^[^ ^2^, ^3^ ^]^ To counter these issues, copper zinc tin sulfide/selenide (CZTS/Se),^[^ ^4^ ^]^ tin sulfide (SnS),^[^ ^5^ ^]^ and antimony sulfoselenide (Sb_2_(S,Se)_3_),^[^ ^6^ ^]^ which encompasses both Sb_2_S_3_ and Sb_2_Se_3_, has emerged as a highly promising light‐harvesting material for PV applications. Over the past few years, notable progress has been achieved in these emerging chalcogenide thin‐film absorbers. For instance, CZTSSe devices have reported efficiencies exceeding ≈14%,^[^ ^4^ ^]^ primarily driven by interface engineering and defect passivation strategies, while CZTS solar cells have exhibited over 13% PCE via gradient bandgap engineering.^[^ ^7^ ^]^ Sb_2_Se_3_ and Sb_2_S_3_ solar cells have also achieved PCEs above ≈10% and ≈8% through crystallographic orientation control and heterojunction optimization,^[^ ^8^, ^9^ ^]^ respectively. SnS‐based solar cells, although still limited to below ≈5% efficiency,^[^ ^10^ ^]^ are actively being explored with compositional engineering and defect management approaches. These advances in the broader chalcogenide family underline the great potential of low‐cost and environmentally friendly absorbers for next‐generation photovoltaics. To be more specific, Sb_2_(S,Se)_3_ rose is a more potential alternative, benefiting from its high absorption coefficient, superior stability, and recognized carrier mobility. Its tunable bandgap (1.1˗1.7 eV), dependent on its Se/S ratio, makes it a foreseeable candidate based on Shockley–Queisser (SQ) limit to achieve over 30% PCE.^[^ ^11^ ^]^ Thus, huge attention has been paid to understanding the fundamentals of this material, including surface quality, grain density/distribution, elemental composition, and interfacial defects.

Till now, various synthetic routes have been developed to explore the performance of Sb_2_(S,Se)_3_ solar cells i.e., spin coating, spray pyrolysis, thermal evaporation, chemical bath deposition (CBD), and hydrothermal deposition (HTD),^[^ ^12^, ^13^, ^14^, ^15^, ^16^, ^17^ ^]^ Recently, water‐rich environment‐based synthesis methods, including chemical bath deposition and hydrothermal processes have reported significant breakthroughs, propelling rapid advancements toward achieving efficiencies exceeding or near 10%.^[^ ^6^, ^15^ ^]^ Most of the top‐performing reports in literature have emphasized on the preferred vertical/tilted (hk1) orientations by acknowledging the anisotropic nature of the material.^[^ ^18^ ^]^ Nevertheless, the progress of this auspicious material is worth watching; the highest PCE to date is far behind its expectation. The main attributes to this suboptimal performance are the complex reaction kinetics, detrimental interfacial defects, and poorly controlled crystalline orientation, having a huge impact on the exciton generation and charge transportation.^[^ ^6^, ^19^ ^]^

To improve the quality of the Sb_2_(S,Se)_3_ film and the device performance, extrinsic doping is an effective strategy. Basically, most of the solution‐processed strategies led to the formation of Sb‐rich Sb_2_(S,Se)_3_ films,^[^ ^20^ ^]^ which are liable to form antisite (Sb_S/Se_) defects, vacancy defects (V_S/Se_), and interstitial defects (Sb_i_). These characteristic defects generate deep electronic levels near the Fermi level, thus acting as provocative forces in charge carrier extraction and transport, ultimately triggering the non‐radiative recombination losses.^[^ ^11^, ^21^ ^]^ To mitigate these detrimental defects and improve key photovoltaic parameters, various doping strategies have been explored. For example, Zhang et al. introduced CsI doping in hydrothermally deposited Sb_2_(S,Se)_3_ to regulate the grain morphology and valence band to improve the charge transfer mechanism, thereby achieving a PCE of 10%.^[^ ^22^ ^]^ Recently, iodide ions were also introduced via surface post‐treatment of Sb_2_(S,Se)_3_ to inhibit the Sb antisite defect to improve the heterojunction quality, delivering a PCE of over 9%.^[^ ^23^ ^]^ Furthermore, Ren et al. adopted a heterogeneous nucleation strategy using BaBr_2_ in the precursor solution to alter the Se/S ratio through Br ion occupation.^[^ ^6^ ^]^ The coordination of the Br with S/Se at the grain boundaries minimized the grain boundary defects, thus improving the PCE from 7.70% to 10.12%. In addition to doping, post‐treatment of the absorber layer has also demonstrated improved crystallinity and optoelectronic properties. For example, sodium fluoride (NaF) and potassium fluoride (KF) have been used to etch the surface of Sb_2_(S,Se)_3_ films.^[^ ^24^, ^25^ ^]^ Both of these materials were reported to optimize the S/Se ratio of the absorber film, which improved the morphology and crystal orientation, hence reporting 16% and 14% increments in the device performance (compared to control devices) for NaF and KF post‐treatments, respectively. Theoretical calculations demonstrated that the cation–anion antisite defects have lower formation energies and are located at the middle levels of the bandgap, prone to recombination centers.^[^ ^26^ ^]^ Hence, surface treatment of the absorber layer may not be sufficient to alter the characteristics of the entire thin film, particularly at the heterojunction.

Alongside absorber quality enhancement, the modification of the heterojunction is the key to advancing the performance of a solar cell. This includes improving the conductivity of the n‐type electron transport layer via doping,^[^ ^16^ ^]^ optimizing the cliff‐like or spike‐like structure at the heterojunction,^[^ ^27^ ^]^ and/or utilizing wide‐bandgap ETL materials to facilitate light capturing and charge extraction.^[^ ^28^ ^]^ However, tuning the absorber layer quality and optimizing the ETL characteristic are two different strategies. For instance, some studies have enhanced the electronic properties of the ETL through targeted doping and surface treatments,^[^ ^29^ ^]^ while others have focused on defect passivation in the absorber layer to suppress non‐radiative recombination.^[^ ^23^ ^]^ Herein, we propose a unified strategy capable of concurrently improving absorber quality and tuning ETL characteristics. A thin layer of KI is spin‐coated between the CdS and Sb_2_(S,Se)_3_ layers. Upon annealing, iodide ions primarily thermally diffuse into the Sb_2_(S,Se)_3_ film, promoting grain growth, improved crystallization, and an elevated work function. Simultaneously, the KI surface treatment improves the conductivity of CdS and tunes its energy band positions. As a result, a favorable spike‐like band alignment is formed at the heterojunction, effectively suppressing interfacial carrier recombination.

## 2 Results and Discussion

### 2.1 Optimization of the KI Treatment Process for CdS Film

**Figure 1:**
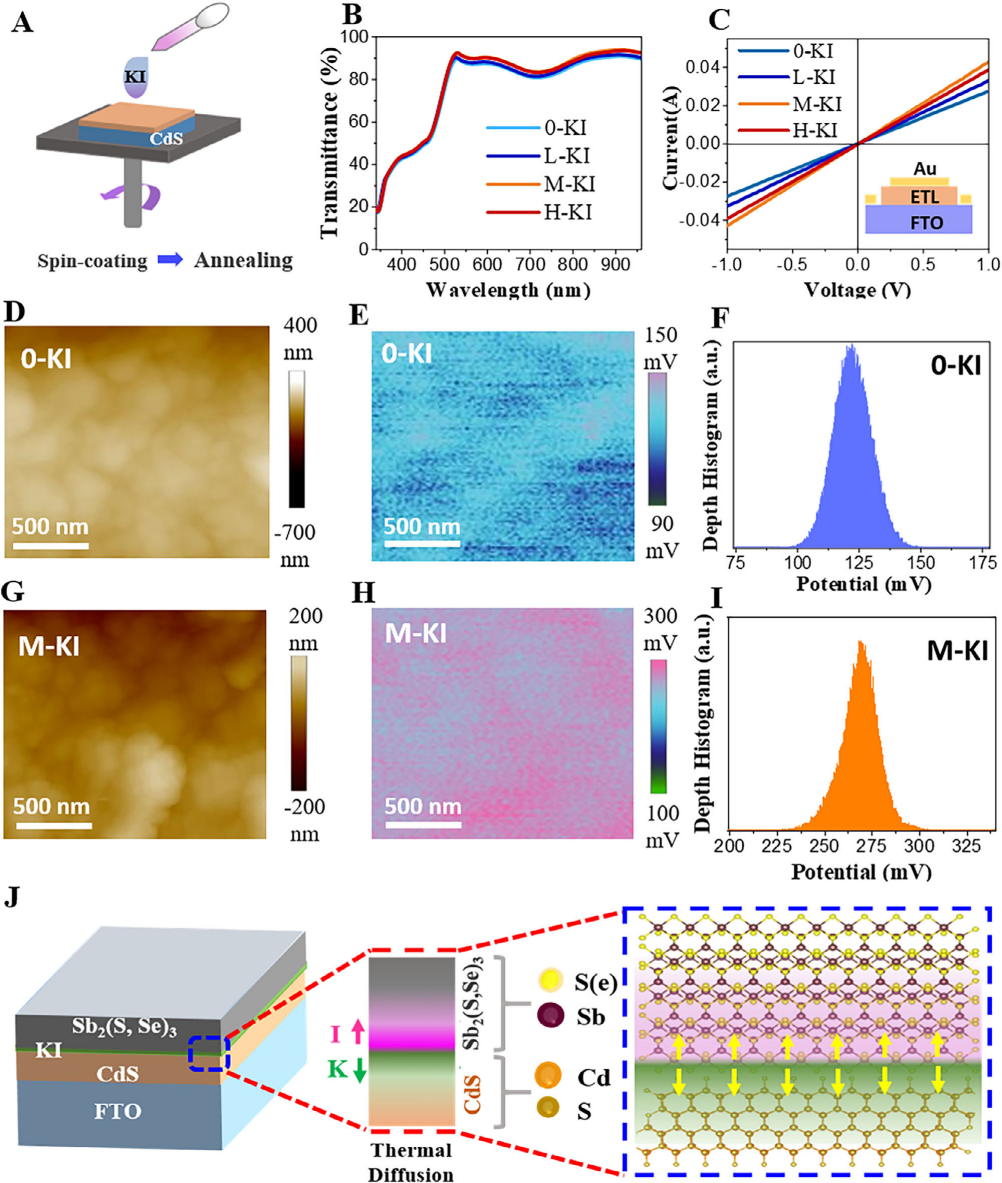
A) CdS treatment with KI, followed by open‐air annealing. B,C) Transmittance spectra, and dark *I‐V* curves for the CdS film treated with different concentrations of KI. D–F) AFM image, KPFM image, and surface potential curve extracted from KPFM for pure and G–I) KI treated CdS films. J) Anticipated diffusion mechanism representation of the KI upon annealing.

**Figure** **1**A demonstrates the CdS film treatment with KI solution. A simple spin coating technique is used to deposit a layer of KI solution of various concentrations (0.03, 0.06, and 0.09 mg ml^−1^), followed by heating at 110 °C. For simplicity, the films treated with different concentrations are labeled as low (L‐KI), medium (M‐KI), and high (H‐KI), while the untreated sample is referred to as 0‐KI. It is important to note that after deposition of the absorber layer on the KI‐treated CdS film (pre‐heated at 110 °C), the CdS/KI/absorber stack undergoes a high‐temperature annealing process at 360 °C. The actual behavior of KI on CdS and its subsequent influence on the absorber can only be reliably assessed under realistic processing conditions. Therefore, to investigate the intrinsic role of KI on CdS, we subjected the KI‐treated CdS film to the same high‐temperature annealing and carefully analyzed its effects. To optimize the KI concentration, we initially studied the transmittance spectrum of the CdS film (Figure 1B). A gradual increase in the transmittance (T %) can be witnessed in the overall wavelength range by increasing the KI concentration. The enhancement in the T% can be helpful in light harvesting efficiency of the absorber layer, ultimately boosting the current density (*J_SC_*) of the device. To investigate the conductivity of the CdS film, the pure CdS film and treated with KI were sandwiched between FTO and Au, and the current voltage curves were obtained under dark conditions, as depicted in Figure 1C. The KI‐based films showed an obvious improvement in the conductivity, increasing from 0.041 for 0‐KI to 0.058 Sm^−1^ for H‐KI. The M‐KI was chosen to be the best, based on its transmittance, conductivity, and PCE of the device, which will be discussed in the latter part of this manuscript. To further clarify the role of KI‐treatment, we investigated the crystal structure and morphology of the CdS film. The X‐ray diffraction patterns of all the devices showed identical peaks with slightly higher intensities for KI‐treatments (Figure S1, Supporting Information). The improved crystallinity not only contributes to the better conductivity and transmittance of the film, but it is expected to improve the quality of the post‐deposited absorber layer. The AFM images of the 0‐KI and M‐KI films are presented in Figure 1D,G, which reflect a better surface quality with lower surface roughness for the KI‐treated film. Kelvin probe atomic force microscopy (KPFM) was used to examine the n‐type doping effect of KI treatment by measuring the contact potential difference (CPD) between the tip of the machine and the surface of the sample. The KPFM mappings shown in Figure 1E,F divulge that the surface potential of the M‐KI (270 mV) is higher than 0‐KI (125 mV), signifying reduced work function.^[^ ^16^ ^]^ The augmented conductivity in the n‐type features enhances electron extraction and endorses the development of high‐quality heterojunctions. To begin the analysis of the absorber layer and the role of the KI‐treated CdS film in improving its quality, we first investigate the presence of KI constituent elements on the CdS film. XPS analysis was performed to examine the composition of CdS films following KI deposition. Figure S2A (Supporting Information) presents the XPS spectra of the CdS‐KI film dried at 100 °C, showing clear evidence of K^+^ and I^−^ ions. As seen in Figure S2A,B (Supporting Information), the characteristic peaks for I^−^ are observed at binding energies of 618 eV (3d_5/2_) and at 632 eV (3d_3/2_), while K^+^ shows peaks at 294 eV (2p_3/2_) and at 296.5 eV (2p_1/2_). In contrast, the high‐temperature annealed film reveals only the potassium‐related peak, as shown in Figure S3A–C (Supporting Information). This suggests that I^−^ ions evaporate at elevated temperatures, whereas K remains adsorbed or doped into the CdS film.^[^ ^29^ ^]^ Thus, the rapid evaporation of I^−^ is expected to contribute to its diffusion into the post‐deposited Sb_2_(S,Se)_3_ layers. An estimated schematic of the described phenomenon is shown in Figure 1J, which will be discussed and further substantiated in a subsequent section of this manuscript. Although the presence of I^−^ in the CdS cannot be completely ruled out, its larger ionic size compared to S^2^^−^ makes it unlikely to replace S^2^^−^. Instead, I^−^ may occupy interstitial or surface sites within the CdS film. As shown in Figure S4 (Supporting Information), an enhanced band edge photoluminescence (≈2.27 eV) and an abridged defect‐related photoluminescence (≈1.75 eV), attributed to sulfur‐related vacancies for the KI‐treated film,^[^ ^30^, ^31^ ^]^ indicating lower defect density in the CdS layer.

### 2.2 Evolution of Device Performance and Characterization

**Figure 2:**
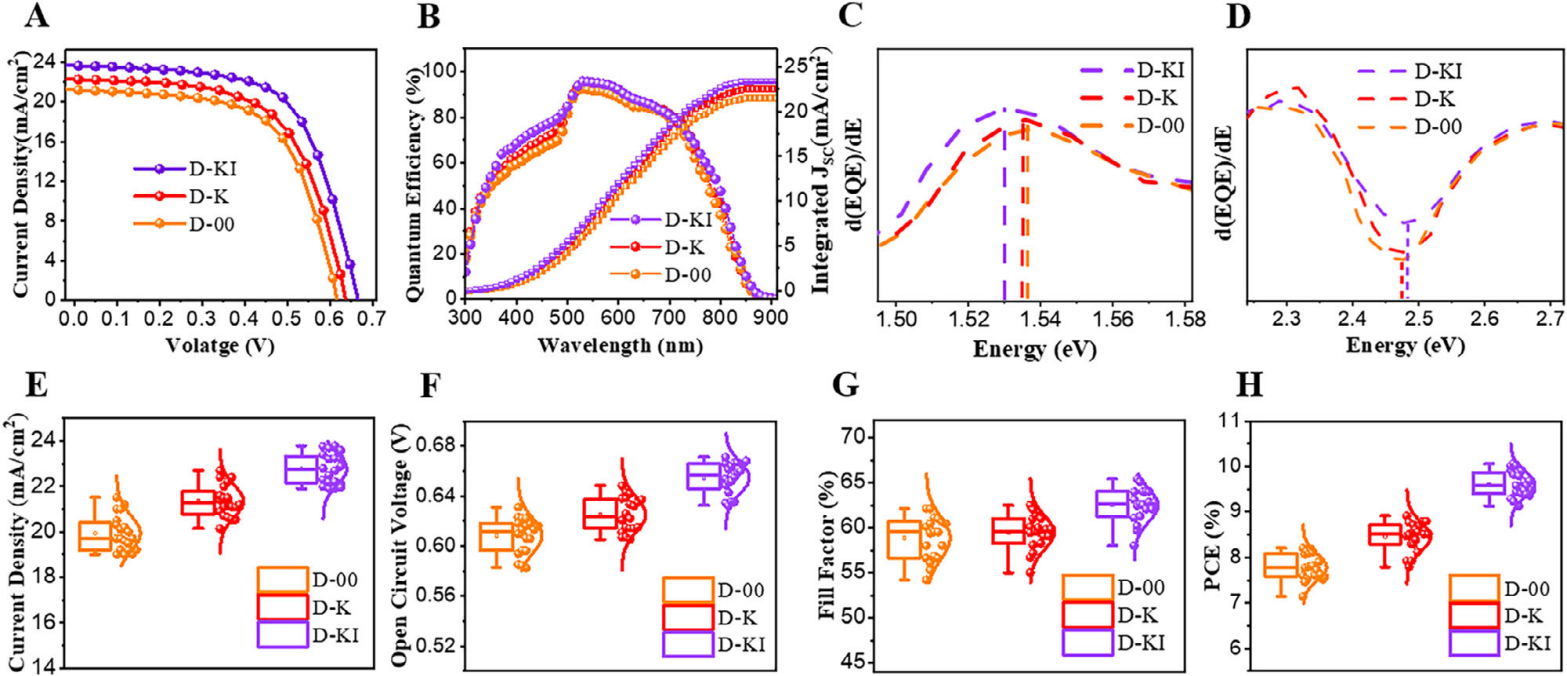
A) Current–Voltage curves and B) EQE spectra for a solar cell based on D‐00, D‐K, and D‐KI films. Extracted bandgaps from the EQE spectra for C) Sb_2_(S,Se)_3_ and D) CdS. E–G) statistical analysis of the solar cell parameters (*J_SC_, FF, V_OC_*, and PCE). A total of 20 devices were analyzed for each measurement.

To evaluate the role of the CdS modification via KI treatment on device performance, we fabricated superstrate‐structured solar cells (FTO/ETL/Sb_2_(S,Se)_3_/Spiro/Au) using three types of ETLs: D‐KI, D‐K, and D‐00. The first sample, labeled D‐KI, uses a CdS film treated with KI but not annealed at high temperature, meaning that both potassium and iodide ions remain in the structure. The second device, D‐K, also uses KI‐treated CdS, but the film was annealed at 400 °C, causing the evaporation of iodide ions, leaving only potassium behind. The third one, D‐00, uses untreated CdS, containing neither K nor I. The current density versus voltage (*J–V*) characteristics of the solar cells are shown in **Figure** **2**A. The performance metrics for these devices are summarized in **Table** **1**. The D‐00 (control) device demonstrated a PCE of 8.13%, with an open‐circuit voltage (*V_OC_*) of 615 mV, a fill factor (*FF*) of 62.42%, and a short‐circuit current density (*J_SC_*) of 21.19 mA cm^−2^. For D‐K device, which features a CdS layer treated with KI and annealed at high temperature, the performance significantly improved. The PCE reached 8.83%, with a *V_OC_* of 635 mV, *J_SC_* of 22.26 mA cm^−^^2^, and a *FF* of 62.45%. The D‐KI device, where KI was deposited on the CdS layer and dried at 110 °C to retain both K and I elements, further enhanced the performance, achieving a PCE of 10.06% with a *V_OC_* of 0.663 V, *J_SC_* of 23.75 mA cm^−^^2^, and *FF* of 63.91%. The evolution in device performance is further confirmed by the statistical box plots shown in Figure 2E–H. The optimization process of the KI‐treatment is also performed to evaluate its effect on the solar cell performance (Figure S5, Supporting Information). At high KI concentration, the device performance remained satisfactory but was more variable and less reproducible. Such fluctuation in the performance can be assigned to uneven distribution of KI, as well as the poor morphology of the absorber film,^[^ ^6^ ^]^ as depicted in Figure S6 (Supporting Information). To further confirm the absorber layer quality improvement and respective solar cell performance enhancement, the effect of KI deposition on the top of the absorber layer was also investigated. The device performance results showed a clear improvement in PCE, increasing from 8.9% for the untreated absorber to 9.66% for the high‐concentration KI‐treated absorber (Figure S7, Supporting Information). Compared to KI incorporation at the CdS/absorber interface, the enhancement was not as pronounced; however, it remained encouraging. This difference can be attributed to the fact that defects at the heterojunction are more severe, so passivation via KI treatment at this interface has a stronger impact on overall device performance. For the low and medium KI concentrations applied to the absorber alone, the PCE improvement was modest, and the device‐to‐device performance variation was higher. The statistical distribution of solar cell performance parameters is depicted in Figure S8 (Supporting Information). Figure 2B presents the external quantum efficiency (EQE) data and the respective integrated *J_SC_*, which align with the *J–V* characterization trends. As expected, fluctuations in EQE values within the 500–800 nm range are observed, reflecting variations in interface and absorber quality. Compared to the control device, both D‐K and D‐KI exhibit improved photo‐response at longer wavelengths, indicating enhanced light‐harvesting capability. Additionally, in the short‐wavelength region, D‐K and D‐KI show a gradual increase in response, contributing to the higher *J_SC_* in these devices. This trend is also consistent with the transmittance spectra of the ETLs. To provide further insight into device performance, the bandgap values of the light absorber and electron transport layers were extracted from the EQE spectra,^[^ ^17^ ^]^ as revealed in Figure 2D,E. The bandgap of CdS increased slightly from 2.47 to 2.48 eV. However, this small change in the CdS bandgap is unlikely to be the main factor behind the enriched device performance, as the enhancement in the EQE is not only in the short wavelength range. While an expected drop in the bandgap of the KI‐treated absorber layer is witnessed.^[^ ^23^ ^]^ The combined improvements in the absorber, CdS, and the interface quality, including better light absorption and charge transport, are responsible for the pragmatic increase in *J_SC_*.

**Table 1:** Performance metrics for D‐00, D‐K, and D‐KI solar cell devices.

Device	PCE (%)	J_SC_ [mA cm^−2^]	V_OC_ (mV)	FF (%)
D‐00	8.14	21.19	615	62.42
D‐K	8.83	22.26	635	62.45
D‐KI	10.06	23.75	663	63.91

### 2.3 Influence of the KI‐Treatment on the Absorber Layer

**Figure 3:**
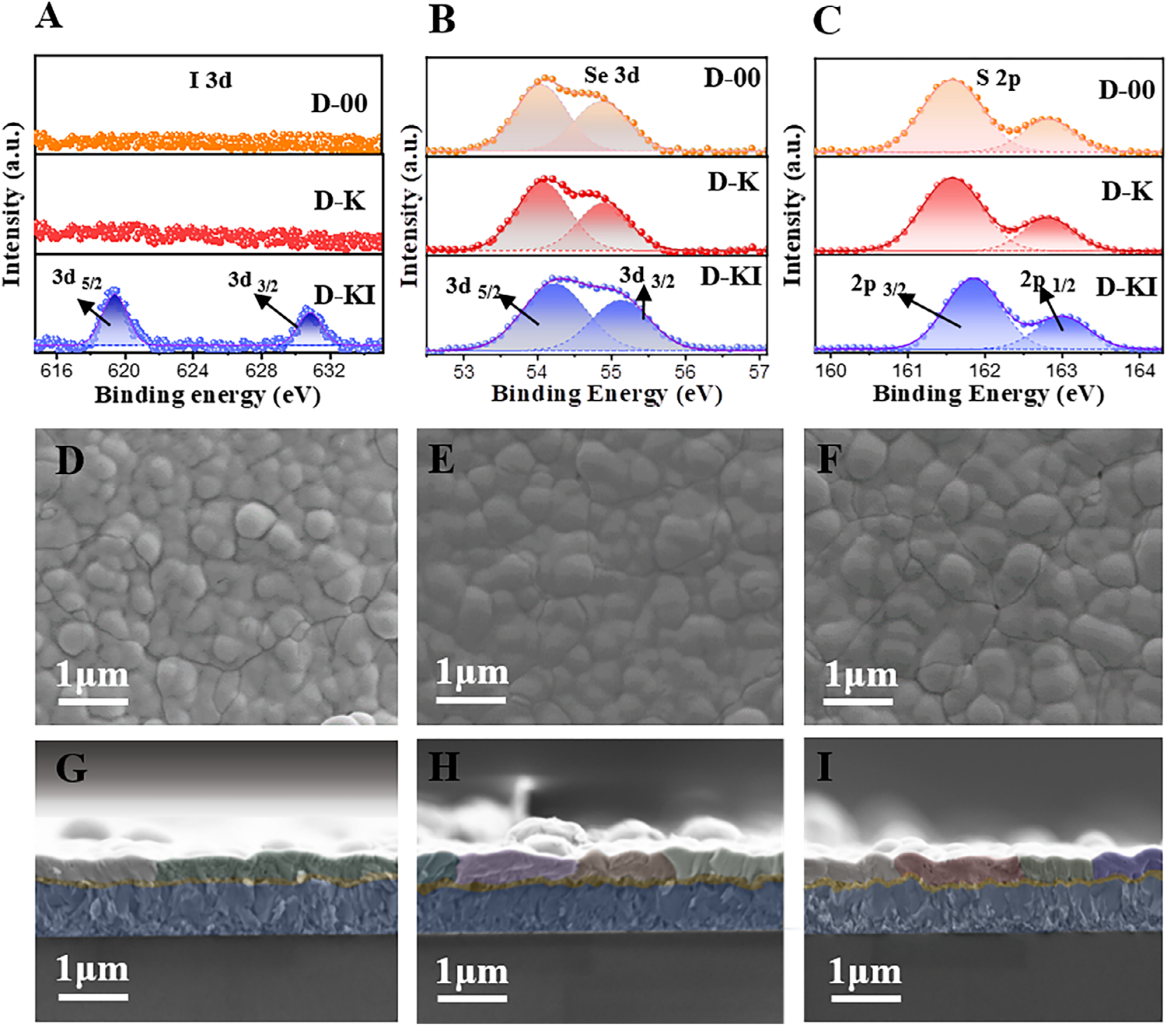
A–C) XPS spectra for I 3d, Se 3d, and S 2p, peaks for absorber films, where the purple, red, and orange colors represent the spectra for D‐KI, D‐K and D‐00 films. D,G) Surface and cross‐cross‐section of K‐00, E,H) for D‐K, and F,I) for D‐KI films.

**Figure 4:**
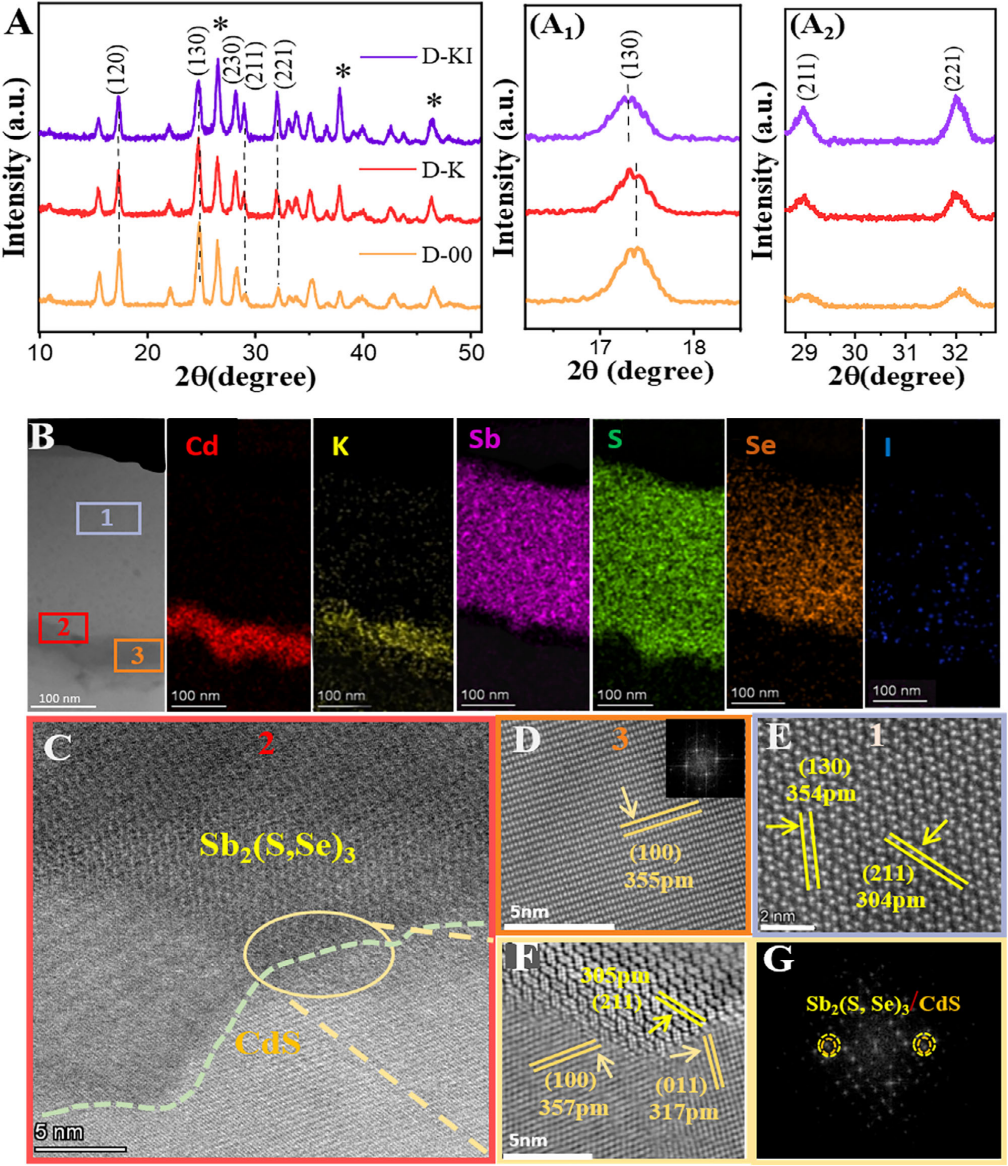
A) XRD patterns for D‐00, D‐K, and D‐KI films, while A_1_, and A_2_ highlight prominent hk0 and kh1 peaks. B) Cross‐section HRTEM image of the cross‐section for D‐KI device, the colored images representing the elemental mapping of the film cross‐section‐wise. C) ETL absorber interface D) CdS‐bulk (E) Sb_2_(S,Se)_3_ bulk, F,G) interface area.

Based on the anticipated preferential diffusion behavior of K^+^ and I^−^ ions (illustrated in Figure 1J and inferred from the structural and compositional analysis of the CdS films presented in the previous section), we first examined the interaction between KI and the absorber layer using X‐ray photoelectron spectroscopy (XPS). To maintain consistency throughout the manuscript, we adopted a uniform naming convention for the Sb_2_(S,Se)_3_ absorber layers deposited on differently treated CdS films and the corresponding complete devices, referring to them as D‐00, D‐K, and D‐KI, respectively. The XPS spectra for I^−^ are depicted in **Figure** **3**A, where the peaks positioned at binding energies of ≈618 and ≈630 eV are assigned to 3d_5/2_ and 3d_3/2_, respectively.^[^ ^23^ ^]^ In agreement with the XPS spectra of the CdS films in Figure S3 (Supporting Information), the signals for iodide ions could not be detected for D‐00 and D‐K samples. In Figure S9 (Supporting Information), the peaks appearing at 530 and 539 eV are related to Sb 3d_5/2_ and 3d_3/2_, correspondingly. Captivatingly, the Sb 3d peaks for D‐00 and D‐K samples are at the same position, as the precursor solution of all the samples contains a fixed amount of potassium in it, while a minute low‐energy shift is visible for D‐KI device. This shift indicates a more electron‐rich local environment for Sb. This effect arises from the interaction between Sb and I^−^, where I^−^ preferentially compensates the excess positive charge of Sb, which is known to introduce deep‐level trap states, rather than merely coordinating with under‐coordinated Sb cations. In this process, I^−^ ions, being negatively charged, can partially donate electron density or compensate for the positive charge associated with Sb antisite defects (a detailed discussion of these defects is provided in the defect analysis section). Consequently, the effective positive charge on Sb atoms is reduced, leading to a decrease in the core‐level binding energy observed in XPS, since the photo‐emitted electrons are less tightly bound. This suggests an interaction between Sb and I ions, implying I^−^ may not only passivate S/Se vacancy and Sb antisite defects,^[^ ^23^ ^]^ and being less electronegative than S or Se, it may alter the local bonding environment around Sb. Furthermore, the high‐energy shift in the binding energies of Se 3d and S 2p in Figure 3B,C signifies the alteration of the absorber layer electronic structure at the atomic level.^[^ ^22^ ^]^ This can be attributed to local charge redistribution in which the incorporation of I^−^ at neighboring Sb sites weakens the covalency of Sb–S/Se bonds, withdrawing electron density from S and Se and thereby modifying their electronic configuration. Thus, the electronic structure modification through I^−^ incorporation in the absorber layer may lead to tuning its energy band alignment, which can sway the charge transfer mechanism at the front and/or back interface.^[^ ^16^ ^]^ It has been established that potassium, including other halides cannot be incorporated in the crystal structure of the antimony sulfoselenide; instead, they may reside on the grain boundaries (GB's).^[^ ^32^ ^]^ Thus, the role of K can influence the grain boundary effect and crystalline orientation. Figure 3D–F highlights the surface morphology of the absorber film deposited on different substrates. The D‐K and D‐KI films exhibit a uniform and even morphology with distinct grain boundaries (GBs), in contrast to the D‐00 film. This difference is further supported by cross‐sectional SEM images. Although the D‐00 film consists of large grains, these grains appear to be aggregates of smaller particles stacked in clusters, as highlighted in green in Figure 3G. In comparison, the D‐K and D‐KI films predominantly feature clear, compact, and large grains, as shown in Figure 3H,I. This suggests that the KI‐treated CdS surface provides better lattice matching, facilitating the lateral growth of the absorber layer. Additionally, the expected diffusion of iodine into Sb_2_(S,Se)_3_ during annealing might be attributed to further enhancing its crystallinity and helps regulate its morphology. The same phenomenon is observed in XRD analysis, as shown in **Figure** **4**A, where we have highlighted the most prominent peaks for the Sb_2_(S,Se)_3_ with dashed lines. A minor shift in the protuberant 130 peak toward lower angle for D‐KI sample in Figure 4A_1_ signifies the incorporation of iodide ion in the crystal structure,^[^ ^22^ ^]^ as the K ion prefers to occupy the grain boundary and may coordinate with the S/Se atoms.^[^ ^32^ ^]^ Furthermore, all the Sb_2_(S,Se)_3_ related peaks showed a gradual growth in the intensities upon increasing the KI‐concentration (Figure S10A,B, Supporting Information). When compared to the D‐00 sample, the D‐K and D‐KI films revealed a gradual growth in vertically oriented (hk1) peaks (Figure 4A_2_), while the intensity of horizontally stacked peaks (hk0) was reduced. The texture coefficient (TC) analysis further confirmed these findings (Figure S11, Supporting Information). For the reference sample (D‐00), the (130) peak exhibits a relatively higher TC value, indicating a preferred orientation along this plane. However, for the D‐K and D‐KI samples, these orientations become less prominent. Concurrently, the TC values for the (hk1) planes increase, approaching a value of one. This trend suggests a shift toward random, but partially vertically oriented crystal growth. The unique 1D crystal structure of Sb_2_(S,Se)_3_, where charge carriers preferentially transport along the [hk1] orientation and hop between (Sb_4_(S,Se)_6_)_n_ ribbons held together by van der Waals forces,^[^ ^27^ ^]^ resulting in more efficient carrier transport in the D‐KI film compared to the control film.

To explore the interface properties, grain orientation, and crystal structure of Sb_2_(S, Se)_3_, transmission electron microscopy (TEM) and energy dispersive spectroscopy (EDS) were employed, driven by compelling findings from the crystal structure and composition analysis of the D‐KI film via XRD and XPS. Figure 4B presents the cross‐section image of the D‐KI film, underlining a compactly adherent and pinhole‐free heterojunction. Region 1, 2, and 3 are marked for Sb_2_(S,Se)_3_, absorber/ETL interface, and for CdS layer, respectively. The subsequent EDS images in Figure 4B revealed divergent contrast for various constituent elements, i.e., Cd, K, Sb, S, Se, and I. Although potassium is incorporated in the precursor solution, it can be initially overlooked here, however, its intense contrast at the CdS surface can be attributed to the fact that it has modified the surface of the CdS and may also be partially doped into it. While I^−^ diffusion in the absorber layer is obvious from EDS mapping, which is consistent with previously discussed XRD and XPS results. The elevated temperatures enhance iodine's diffusion kinetics, allowing it to move into the Sb_2_(S,Se)_3_ from the CdS layer. The grain boundaries and/or defective regions in Sb_2_(S,Se)_3_ act as favorable pathways for iodine diffusion, which improves the electronic properties of the absorber, enhancing carrier collection and potentially boosting device efficiency. The lattice‐fringe high‐resolution scanning transmission electron microscopic (STEM) images assimilated for absorber/CdS interface, CdS layer, and Sb_2_(S,Se)_3_ layer are presented in Figure 4C–E. The targeted bulk area of the CdS revealed a prominent 355 pm wide lattice attributed to the (100) planes, while Sb_2_(S,Se)_3_ revealed interplanar d‐spacings of 304 pm and 354 pm, corresponding to the (211) and (130) planes.^[^ ^33^ ^]^ Figure 4F exhibits a high‐resolution TEM (HRTEM) image of a specific region of the CdS/Sb_2_(S,Se)_3_ heterojunction highlighted by the yellow circle in Figure 4C, where the fast Fourier transformation (FFT) patterns of the CdS and Sb_2_(S, Se)_3_ show a clear overlap, suggesting preferred lattice matching (Figure 4G). Interestingly, the interface area revealed a different lattice fringe of 317 pm for the CdS‐(011) plane and 305 pm for the absorber layer (211) plane. Compared to pure CdS, the d‐spacing of the (100) and (011) planes is slightly wide,^[^ ^33^ ^]^ which can be assigned to the K^+^ ion occupying the most likely octahedral interstitial sites in the CdS lattice. The coherent lattice fringes formed at the interface without any obvious dislocation, and the minimum lattice mismatch signifies the emergence of preferentially (kh1) oriented growth of the Sb_2_(S,Se)_3_ on the CdS surface.^[^ ^6^ ^]^

**Figure 5:**
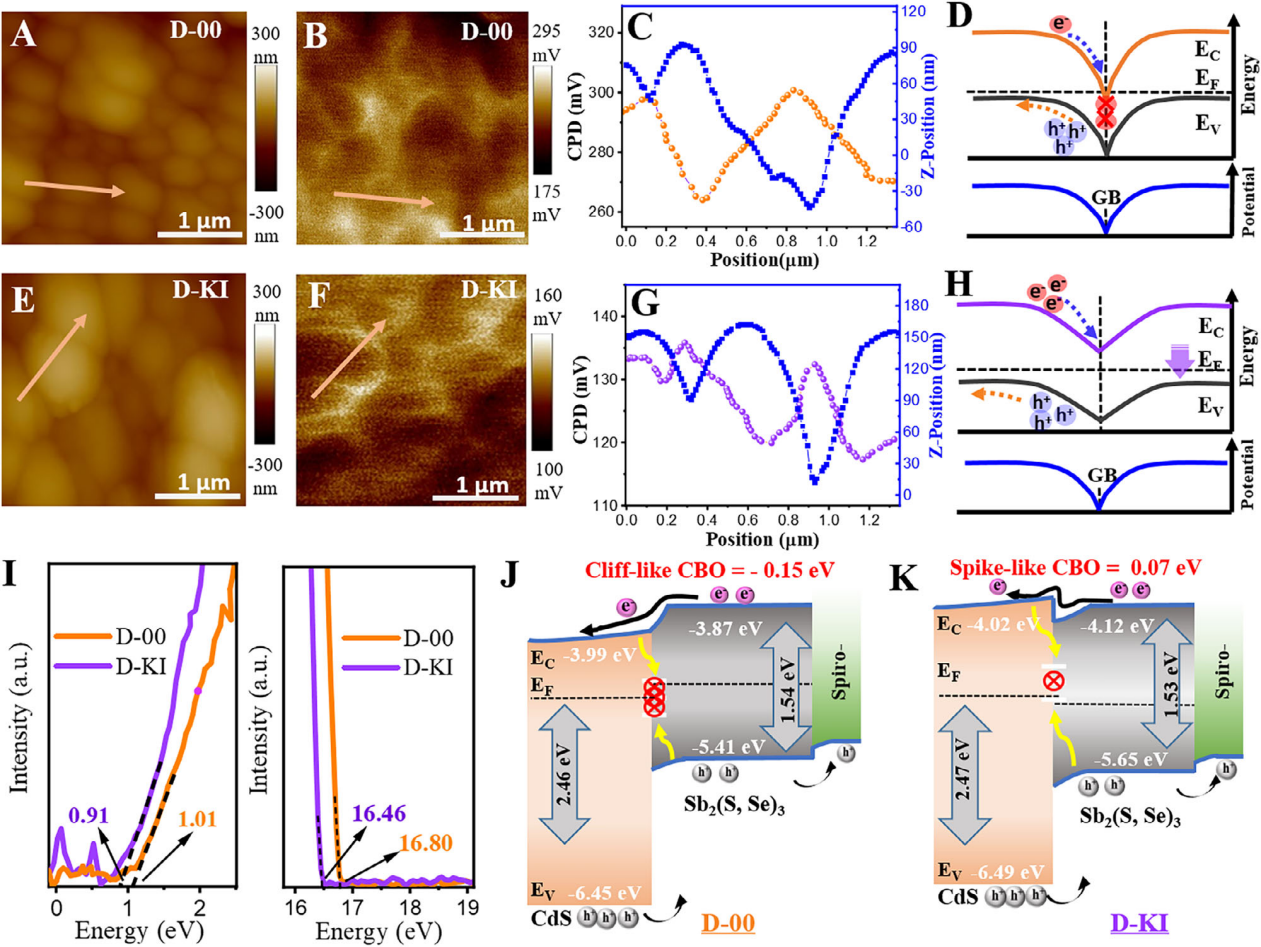
A,B) AFM and KPFM images of Sb_2_(S,Se)_3_ films deposited on pure CdS film (K‐00) and E,F) KI‐treated CdS films (D‐KI). C,G) The corresponding topography and potential line scans extracted from AFM and KPFM images. (D,H) Schematic illustration of band diagram and CPD near the GBs. I) Ultraviolet photoelectron spectroscopy for both devices. J,K) Schematic illustration of carrier transfer in the Sb_2_(S,Se)_3_ devices without and with KI treatments.

It is worth noting that we focused our comparison on the D‐00 and D‐KI samples to better isolate and understand the effects arising from the combined presence of K^+^ and I^−^. Notably, the D‐KI films exhibited distinct differences in structure, morphology, and crystallinity compared to the untreated D‐00 films, while it partially resemble D‐K in certain aspects. To avoid confounding effects from the shared presence of K^+^, we focused our subsequent investigation on comparing D‐00 and D‐KI. This approach offers a clearer view of the specific impact of KI surface treatment and its potential for enhancing the performance of Sb‐chalcogenide solar cells, in line with the core objective of this study. To distinguish the surface potential and understand the influence of iodide incorporation in the absorber layer, Kelvin probe atomic force microscopy (KPFM) was conducted (**Figure** **5**). The atomic force microscopy (AFM) images also supported the SEM findings, as R_avg_ of the D‐KI was found to be much lower (27.5 nm) than the control film (37.8 nm), supplemented by an upsurge in grain size and uniformity. This reduction in roughness and enhancement in grain structure are beneficial for achieving a high‐quality heterojunction interface.^[^ ^6^ ^]^ From KPFM analysis in Figure 5B,F, the surface potential (SP) is determined by applying the bias voltage to the samples, where the following equation is applied to extract the information.^[^ ^34^ ^]^

(1) $$\varphi_{\mathrm{SP}}=\varphi_{\mathrm{PP}}-qV_{\mathrm{CPD}}$$

where 𝜑_SP_ is the sample potential, 𝜑_PP_ is the applied potential to the probe, q is the elementary charge, which is usually termed as e^−^ in such equations, and V_CPD_ is the contact potential difference. The area marked by the arrow is scanned to extract the information about the topography and potential profiles. The scanned range of the surface potential for the control and D‐KI devices was 17 to 295 mV and 100 to 160 mV, respectively. It is witnessed in Figure 5C,G that the contact potential difference (V_CPD_) between the grain interior (GI) and grain boundary (GB) is not the same for both types of films. The surface potential of the D‐KI film was comparatively lower (Figure S12, Supporting Information). As the scanning probe goes from the grain surface to the grain boundary, the potential gets higher. Precisely, the average V_CPD_ (V_GB(CPD)_‐V_(GI)CPD_) of D‐00 film is 32 mV, which is higher than the one for D‐KI film (17.6 mV), signifying an elevated work function (WF) for the D‐KI sample.^[^ ^35^ ^]^ The variation in the work function can be associated to the fluctuation in the Fermi level.^[^ ^36^ ^]^ A higher WF designates a downward shift in the fermi level for D‐KI films, as described in Figure 5H, which can elevate the hole concentration of the material.^[^ ^6^ ^]^ In summary, the deepened Fermi level is prone to generate a benevolent band bending to expedite carrier separation.^[^ ^37^ ^]^ Additionally, the lower V_CPD_ can be attributed to a subtle yet more favorable band bending at the grain boundaries in the D‐KI sample.^[^ ^38^ ^]^ This beneficial band bending helps prevent electron trapping by defects at the grain boundaries, thereby enhancing charge carrier transport and collection, while minimizing carrier recombination. The schematics of the discussed mechanism are portrayed in Figure 5D,H.

With the confirmed incorporation of iodide ions into the crystal structure of the absorber layer and surface modification of the CdS via KI‐treatment, the optoelectronic properties have been significantly altered. These modifications are also anticipated to impact the energy levels of the materials. Consequently, the energy band structure of Sb_2_(S,Se)_3_ films and their alignment with the electron transport layers (ETLs) were investigated using ultraviolet photoelectron spectroscopy (UPS) (Figure 5I), which provides clear insights into the carrier separation and transfer mechanisms within the device. The UPS spectra for the pure CdS and KI‐treated CdS are presented in Figure S13 (Supporting Information). The Fermi level (E_F_), Valence band energy (E_V_), and conduction band positions (E_C_) were measured according to the literature, and the details are provided in Note 2 (Supporting Information). As expected, the CdS exhibited a negligible variation in its conductivity type, however, the E_C_ slightly moved up from −4.00 to −4.02 eV. On the other hand, the E_F_ of the absorber layer dropped from −4.40 to −4.74 eV, while the E_C_ moved from −3.87 to −4.12 eV, respectively. Based on the extracted energy level positions from the UPS analysis, an energy band diagram is provided to clearly illustrate the charge transfer mechanism. Agreeing to the literature that p‐orbitals of the S and Se are associated to the valance band of the antimony sulfoselenide materials,^[^ ^39^ ^]^ we believe that the diffusion of iodide ions not only occupies some defect sites in the absorber film, but also alters the S to Se ratio as well.^[^ ^6^ ^]^ That is why a vital change in the *E_V_* is observed, which ultimately influenced the positions of *E_C_*. The UPS revealed both the films to be n‐type semiconductors; however, a minor downshift of the *E_F_* for D‐KI film reflects comparatively more prominent hole concentration, which is also consistent with the observed reduction in surface potential. The conduction band offset (CBO) is a vital characteristic to define the carrier transfer and separation at a heterojunction, which are typically classified into spike‐like and cliff‐like structures.^[^ ^27^ ^]^ Interestingly, the cliff‐like CBO (−0.15 eV) of the D‐00 transformed to a spike‐like CBO of 0.07 eV for D‐KI device (Figure 5J,K). The cliff‐like band alignment is reported to be detrimental for charge recombination at the interface, reducing carrier lifetime, which eventually results in a substantial loss in the *V_OC_* of the device, as portrayed in Figure 5J. Thus, an optimal spike is indicative of the moderate interface recombination with slight notch development. Consequently, KI‐treatment efficiently normalizes the band alignment and passivates the interface recombination.

### 2.4 Heterojunction Interface, Carrier Dynamics, and Defects Analysis

**Figure 6:**
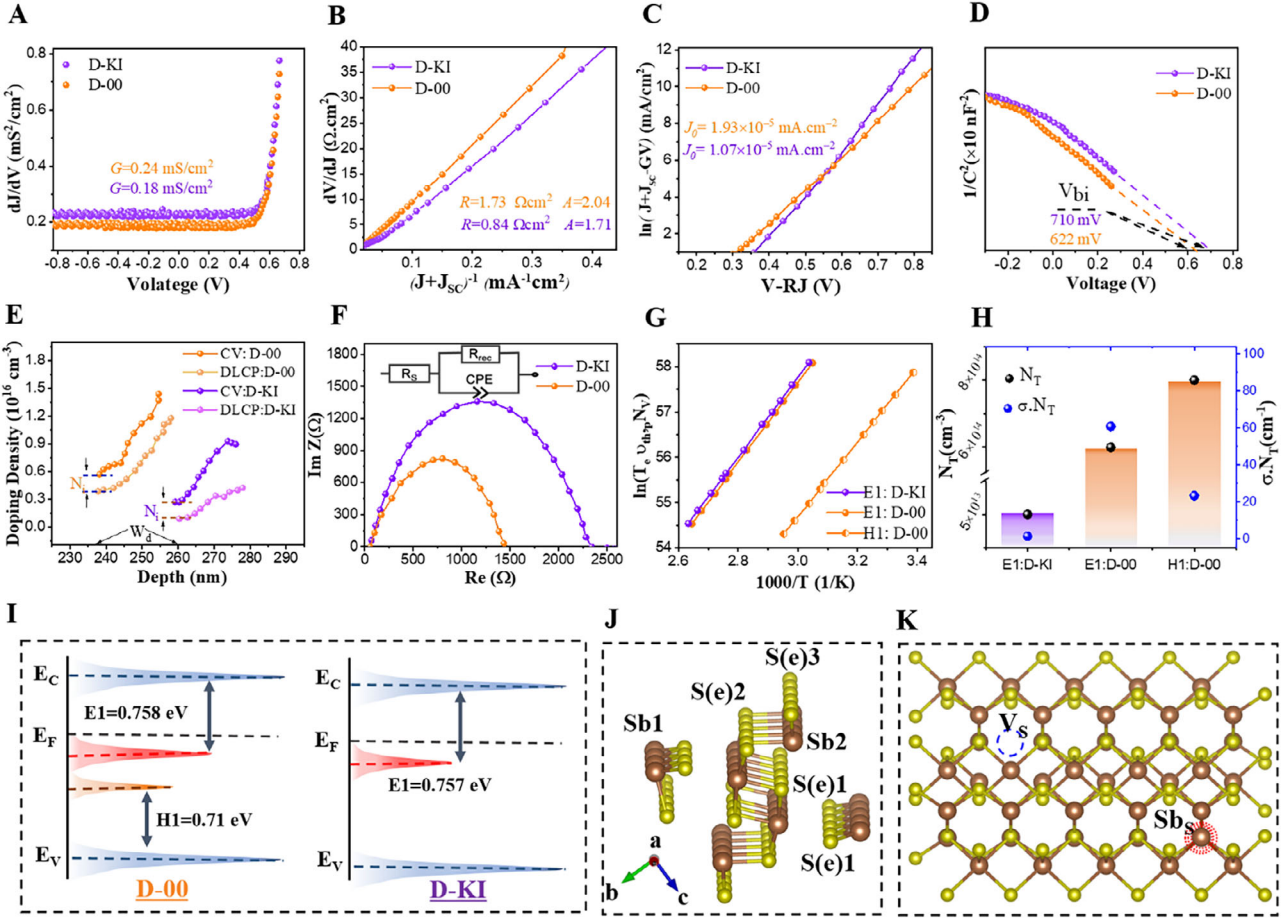
A) The characterization of Parallel conductance G. B) Series resistance (R) and ideality factor. C) Reverse saturation current density (*J*_0_) characterizations. D) C^−2^ against V curves for built‐in potential calculation. E) *C–V* and DLCP curves for N_i_ and W_d_ extraction, F) Nyquist plots of Sb_2_(S,Se)_3_ devices with KI and without KI modifications to calculate recombination resistance, and the equivalent circuit diagram is presented inside the figure. G,H) Arrhenius plots attained from DLTS signals and the extracted defect density and capture cross‐section analysis of the corresponding devices. I) Energy levels and defect levels of the control (D‐00) and champion (D‐KI) devices. J) Crystal structure and atomic sites in the ribbon of Sb_2_(S,Se)_3_. K) Representation of defect formation and proposed suppression in the crystal structure.

To comprehend the outstanding performance of D‐KI device, we analyzed the series resistance (*R_S_*), ideality factor (*A*), and reverse saturation current (*J_0_*) derived from the mathematical treatment of the dark *J–V* curves of the respective devices,^[^ ^40^ ^]^ as explained in supporting information Note 1, and summarized in **Table** **2**. The *dJ/dV* versus *V, dV/dJ* versus (*J_SC_‐J)^−1^*, and (*J+J_SC_‐GV*) versus *V‐RJ* curves were used to obtain the insights of the heterojunction of the devices, as shown in **Figure** **6**A–C. The *G* standards for D‐00 and D‐KI devices were 0.24 and 0.18 mS cm^−2^, respectively, while the respective *R_S_* values were calculated to be 1.73 Ω cm^2^ and 0.84 Ω cm^2^, as shown in Figure 6B. This also signifies an increased shunt resistance for D‐KI device. The ideality factor (*A*) and reverse saturation current (*J_0_*) significantly reduced from 2.04 to 1.71 and 1.93 × 10^−5^ to 1.07 × 10^−5^ mA cm^−2^ benefiting from the reduced loss of photo‐generated charge carriers in the thin absorber film and its interface with the electron transport layer.^[^ ^41^ ^]^ In addition, the better *A* values for D‐KI designate that carrier recombination at the GB and the back surface can be effectively suppressed, which can be attributed to the partial incorporation of iodide ions at GBs as well. To uncover the KI role, we further explored the photoelectric and electrochemical tests of the control and modified devices. The built‐in potential (*V_bi_*) of KI based device showed an obvious enhancement from 622 mV to 710 mV (Figure 6D). This boost in the *V_b_*_i_ can be assigned to a higher open circuit voltage and minimized diode current.^[^ ^34^ ^]^ The interface quality of both devices was further evaluated by coupling capacitance–voltage (*C–V*) with drive‐level capacitance profiling (DLCP), as shown in Figure 6E. The interface defect density (*N_i_*) of the device can be reduced from 0.4 × 10^16^ cm^−3^ to 0.2 × 10^16^ cm^−3^ upon KI treatment. In addition, the enhanced D‐KI heterojunction shows a wider depletion width (W_d_) of 258 nm at V = 0 V, compared to the control device, which exhibited a W_d_ of 238 nm. This indicates improved performance of the D‐KI device, with better collection and extraction of charge carriers. This suggests that the detrimental defects in Sb_2_(S,Se)_3_ can be effectively suppressed by iodine diffusion through the KI treatment, while the modified CdS interface offers better lattice matching, promoting improved growth mechanisms and crystallographic orientations. This enhanced interface is expected to reduce undesirable band alignment near the surface of the Sb_2_(S,Se)_3_ thin film, making it less likely for carriers to be trapped by defects. As a result, carriers can be more easily transported at the interface, leading to increased *J_SC_* and *FF* of the device. To further investigate the kinetics of interfacial charge transport in the D‐00 and D‐KI devices, electrochemical impedance spectroscopy (EIS) was performed, where the obtained results are depicted in Figure 6F. The fitting results reveal that the recombination resistance (*R_rec_*) increased from 1480 Ω for the D‐00 device to 2326 Ω for the D‐KI device. This noteworthy increase in resistance underscores the improved carrier transport and reduced trap‐assisted recombination in the optimized D‐KI device.

**Table 2:** A summary of the interface characteristics of D‐00 and D‐KI devices.

Device	G [mS cm^−2^]	R (Ω.cm^2^)	A	J_0_ [mA cm^−2^]	V_bi_ (mV)	N_i_ [cm^−3^]	W_d_ (nm)
D‐00	0.24	1.73	2.04	1.93 × 10^−5^	622	0.4 × 10^16^	238
D‐KI	0.18	0.84	1.71	1.07 × 10^−5^	710	0.2 × 10^16^	258

To understand the nature of defects and the influence of KI incorporation on defect states within the bulk Sb_2_(S,Se)_3_, deep‐level transient spectroscopy (DLTS) was employed. As shown in Figure S14A (Supporting Information), temperature‐dependent transient capacitance measurements revealed one electron trap‐related peak and one hole trap‐related valley in the control (D‐00) device. While in D‐KI sample exhibited only one electron trap, indicating that one of the original trap states was eliminated upon KI‐treatment (Figure S14B, Supporting Information). Based on the n‐type nature of Sb_2_(S,Se)_3_, the traps were identified as an electron trap (E1) and a hole trap (H1), with H1 being particularly significant. The Arrhenius analysis (Figure 6G) enabled extraction of the activation energies and capture cross‐sections of these traps, as plotted in Figure 6H, based on standard emission rate equations below: where τ, υ_th_, σ, and N_(c, v)_ represents emission time, thermal velocity, effective state density for the conduction band (c) and valence band (v), while the n and p in the subscripts are the indicatives of electron and hole, respectively.

(2) $$\ln\left(\tau_{n}\upsilon_{th,n}N_{c}\right)=\left(\frac{E_{c}-E_{T}}{k}\frac{1}{T}-\ln\left(X_{n}\sigma_{n}\right)\right)$$

(3) $$\ln\left(\tau_{p}\upsilon_{th,p}N_{V}\right)=\left(\frac{E_{T}-E_{V}}{k}\frac{1}{T}-\ln\left(X_{p}\sigma_{p}\right)\right)$$

*Х* is the entropy factor for electron (n) and hole (p), while the term E_T_–E_V_ (E_C_–E_T_) highlights the location of the trap states in the bandgap, extracted from the slope of the equation. The observed defects characteristic via DLTS analysis are summarized in **Table** **3**. It is important to highlight that E1 trap states exhibited comparable activation energies of 0.757 and 0.758 eV in both D‐00 and D‐KI devices, suggesting that they likely originate from the same categories of intrinsic defects. E1 traps are electron states below E_C_, attributed to vacancy defect (V_S/Se_).^[^ ^17^, ^42^ ^]^ H1 is hole trap states above *E_V_* in D‐00, and they are associated with antisite defects, where the Sb atoms substitute Se/S atoms at different lattice sites (denoted as Sb_(Se/S2)_ and Sb_(Se/S)1_).^[^ ^16^, ^18^, ^20^ ^]^ Interestingly, the H1 state completely disappeared in D‐KI device, signifying the complete suppression of antisite defects in it. On the other hand, the defect density (N_T_) of E1 significantly reduced from 8.80 × 10^14^ cm^−3^ to 5.73 × 10^13^ cm^−3^ in D‐KI device. This improvement is attributed to iodine diffusion into the Sb_2_(S,Se)_3_ lattice, where I^−^ ions preferentially occupy S or Se vacancy sites, thereby reducing the likelihood of these defects. Thus, the introduction of I^−^ ions helps compensate for these vacancies, thereby suppressing the formation of such deep‐level defects. Figure 6I–K presents the defect energy levels, their corresponding density distributions, and the defect realization mechanism. These energy positions were determined using previously discussed ultraviolet photoelectron spectroscopy coupled with the bandgap information of Sb_2_(S,Se)_3_. Among the identified traps, H1 was found to lie closest to the Fermi level and exhibited the largest capture cross‐section (9.4 × 10^−14^ cm^2^), leading to Fermi level pinning: a phenomenon known to hinder device efficiency.^[^ ^43^ ^]^ Furthermore, the carrier lifetime of the films was evaluated using the (𝜎×N_T_) values. For the E1 trap in the D‐KI sample, the (𝜎×N_T_) value is the lowest (1.295 cm^−1^), indicating a longer carrier lifetime. This suggests that the improved efficiency of the D‐KI device is primarily due to the passivation of H1 traps and the partial suppression of E1 traps.

**Table 3:** A summary of the bulk defects characterized by DLTD for D‐00 and D‐KI devices.

Device	Trap	E_T_ (eV)	σ(cm^2^)	N_T_ [cm^−3^]	σ×N_T_ [cm^−1^]
D‐00	H1	0.710	9.41 × 10^−14^	6.47 × 10^14^	60.88
	E1	0.758	2.64 × 10^−14^	8.80 × 10^14^	23.23
D‐KI	E1	0.757	2.26 × 10^−14^	5.73 × 10^13^	1.295

Finally, the time‐resolved photoluminescence (TRPL) analysis was conducted using 470 nm excitation light to analyze the carrier lifetime evaluation with the addition KI (Figure S15, Supporting Information). The details of the measurements are provided in supporting information Note 3. The TRPL analysis reveals that the D‐KI device exhibits a slower average decay (τ_D‐KI_ = 6.91 ns) compared to the control device (τ_D‐00_ = 3.96 ns), signifying an enhancement in the carrier lifetime. This slower decay is attributed to the passivation of defects at the CdS/Sb_2_(S,Se)_3_ interface and within the absorber layer itself.^[^ ^44^ ^]^ The diffusion of iodine into the Sb_2_(S,Se)_3_ film apparently reduces trap states and recombination centers, allowing charge carriers to persist longer before recombining. This improvement in carrier lifetime is consistent with the observed increase in J_SC_ and *FF*, as longer carrier lifetimes facilitate more efficient charge extraction. Additionally, the KI treatment could improve the interface quality and band alignment, further reducing recombination and enhancing device performance.

## 3 Conclusion

In this study, we demonstrated a unified strategy for enhancing the performance of Sb_2_(S,Se)_3_‐based solar cells by simultaneously improving the absorber quality and electron transport layer (ETL) characteristics. The spin‐coating of a thin KI layer between the CdS and Sb_2_(S,Se)_3_ layers, followed by thermal annealing, enabled the diffusion of iodide ions into the absorber, leading to substantial grain growth, improved crystallization, and a higher work function. This treatment effectively eliminated detrimental vacancy defects and significantly reduced electron trap states. Furthermore, the junction quality was markedly improved, as evidenced by a reduction in the ideality factor from 2.04 to 1.71, indicating enhanced carrier extraction and reduced recombination. Additionally, the KI treatment promoted a vertical [hk1] orientation in the absorber, while the undesirable [hk0] orientation was minimized. These synergistic effects resulted in a significant boost in device efficiency, with the champion solar cell achieving a power conversion efficiency (PCE) of 10.06%, surpassing the control device's PCE of 8.14%. Our findings provide a promising approach for optimizing both absorber and ETL characteristics, paving the way for further advancements in Sb_2_(S,Se)_3_‐based photovoltaic devices.

## Conflict of Interest

The authors declare no conflict of interest.

## Supporting information



Supporting Information

Supporting DataFile

## Data Availability

The data that support the findings of this study are available from the corresponding author upon reasonable request.
