# Supplementary material for: Synergistic Passivation of Bulk and Heterojunction Defects via Dual‐Functioned Interlayer Treatment for High‐Efficiency Sb2(S,Se)3 Solar Cells
Source: Adv Sci (Weinh). 2025 Oct 5;12(47):e15777. doi: 10.1002/advs.202515777 (PMC12713091; doi:10.1002/advs.202515777)
Supplement: Supplementary file 1 — Supporting Information [file ADVS-12-e15777-s001.docx]

Supporting Information

Synergistic Passivation of Bulk and Heterojunction Defects via Dual-Functioned Interlayer Treatment for High-Efficiency Sb_2_(S,Se)_3_ Solar Cells

Muhammad Ishaq^1^, Yi-Ming Zhong^1^, Muhammad Abbas^1^，Adil Mansoor^1^_，_Boyang Fu^1^, Jun Zhao^1^, Zhenghua Su^1^， Shuo Chen^1^, Rajwali Khan^2^*, Zhuanghao Zheng^1^* ,Guangxing Liang^1^*

^1^ Shenzhen Key Laboratory of Advanced Thin Films and Applications, Key Laboratory of Optoelectronic Devices and Systems of Ministry of Education and Guangdong Province, State Key Laboratory of Radio Frequency Heterogeneous Integration, College of Physics and Optoelectronic Engineering, Shenzhen University, Shenzhen, Guangdong 518060, China

^2^ National Water and Energy Center, United Arab Emirates University, Al Ain, 15551, United Arab Emirate.

Corresponding Author Email: [rajwali@uaeu.ac.ae](mailto:rajwali@uaeu.ac.ae) (Prof. Khan)

zhengzh@szu.edu.cn (Prof. Zheng)

lgx@szu.edu.cn (Prof. Liang)

**Experimental Section**

*ETL Preparation：*The CdS film was deposited via chemical bath deposition method on chemically cleaned and O_3_ treated FTO substrates. The substrates were immersed for 17 min in a precursor solution of 13 mL thiourea solution (1.5 M), 20 mL Cd(NO_3_)_2_ solution (15 mM), 26 mL ammonium oxide, and 135 ml of deionized water, all maintained at 65 C. After deposition, the CdS film was post-treated by spin coating CdCl_2_/methanol solution (20 mg mL^-1^) for 20s, followed by a subsequent 10 min heat treatment at 400 °C in open air.

*Sb_2_(S,Se)_3_ Deposition by HT method*：The Sb_2_(S,Se)_3_ film was deposited on the substrate through HT method. Initially the 0.50g antimony potassium tartrate (APT) was added in 80 ml D.I water while stirring. When the APT was dissolved, 1.6 g of Na_2_S_2_O_3_ (STS) was added, and the solution was allowed to stir for 5 min. finally 50 mg CH_4_N_2_Se (SU) was added in the solution. Once the SU was completely dissolved, the solution was poured into two 40 mL Teflon containers, each containing angled FTO/CdS samples suspended within. Teflon containers were then sealed in autoclaves and heated at 140 °C for 2 hours and 10 minutes. After naturally cooling to room temperature, the samples were retrieved and dried in a furnace at 70 °C. Finally, the as-deposited films were annealed in a nitrogen-filled glovebox at 360 °C for 15 min. Afterwards, a Spiro-OMeTAD layer was spin-coated onto the films, followed by thermal evaporation of a gold (Au) back contact. For KI treatment, various concentrations of KI powder were dissolved in ethanol using ultra sonication, and the solution was spin-coated onto the CdS layer at 300 rpm for 20 seconds. The treated samples were then dried in ambient air at 110 °C 10 minutes.

*Thin Film and Device Characterizations：*The Structural properties of the synthesized films were examined via X-ray diffraction (XRD, Ultima IV) employing Cu Kα radiation to identify crystallographic phases. Surface elemental composition and chemical states were analyzed using X-ray photoelectron spectroscopy (PHI 5000 Versa Probe II, Ulvac-Phi, Japan) equipped with a monochromatic Al Kα source (1486.7 eV, 50 W, 16 kV, 200 µm beam spot). Due to diffusion gradient of the iodide ion from the interface to the surface, we prepared extremely thin Sb_2_(S,Se)_3_ film for XPS analysis to make sure the iodide detection. Morphological features were visualized using a high-resolution thermal field emission SEM (Zeiss SUPRA 55), while nanoscale surface texture was quantified through atomic force microscopy (NT-MDT), providing RMS roughness values. Cross-sectional transmission electron microscopy (TEM) images were acquired using an FEI Titan Cubed ThemisG2 300 microscope. Sample preparation for TEM was carried out using a focused ion beam system (FEI Scios). Elemental depth profiles were obtained through TEM analysis combined with energy-dispersive X-ray spectroscopy (EDS). The interfacial energy level alignment was explored using ultraviolet photoelectron spectroscopy (UPS, PHI 5000 VersaProbe III) with a He I (21.22 eV) discharge lamp. Optical characteristics, were evaluated using a Shimadzu UV-3600 spectrophotometer integrated with a monochromatic light source.

For device performance assessment, *J–V* curves were recorded under standard AM 1.5G illumination (100 mW/cm²) using a calibrated 3A solar simulator and a Keithley 2400 source meter. The external quantum efficiency (EQE) spectrum was acquired using a Zolix SCS101 setup featuring a monochromator and the same Keithley unit. Capacitance–voltage (*C–V*) measurements were conducted in the dark with a 30 mV AC signal at 10 kHz, sweeping the DC bias from -1 V to 0.3 V. To evaluate thermally activated recombination mechanisms, temperature-dependent open-circuit voltage (*V_OC_*) measurements were performed using a Lakeshore 325 controller within a Janis VPF-100 cryostat cooled by liquid nitrogen, spanning 400 K to 100 K. Electrochemical impedance spectroscopy was carried out under 0.6 V bias using a Zahner Zennium workstation. Capacitance-voltage (*C–V*) and drive-level capacitance profiling (DLCP) measurements were conducted using a Keithley 4200A-SCS system integrated with a JANIS cryogenic stage. Time-resolved photoluminescence (TRPL) analysis was carried out on the devices utilizing a FluoTime 300 spectrograph, excited at a wavelength of 532 nm.

**Statistical Analysis**

For all device performance measurements, at least 10 devices were fabricated and tested for each condition. Photovoltaic parameters including power conversion efficiency (PCE), open-circuit voltage (V_OC_), short-circuit current density (J_SC_), and fill factor (FF) are reported as mean ± standard deviation (SD) to reflect device-to-device variation. Device reproducibility was evaluated, and the variation across multiple devices is provided in the corresponding figures (e.g., Figures 2, Figure S5, and S8). No formal hypothesis testing was applied, as the study focuses on comparative device performance under different KI treatment conditions. The statistical data provided ensure the reliability and reproducibility of the reported improvements in device efficiency and allow readers to assess the significance of the observed trends

**
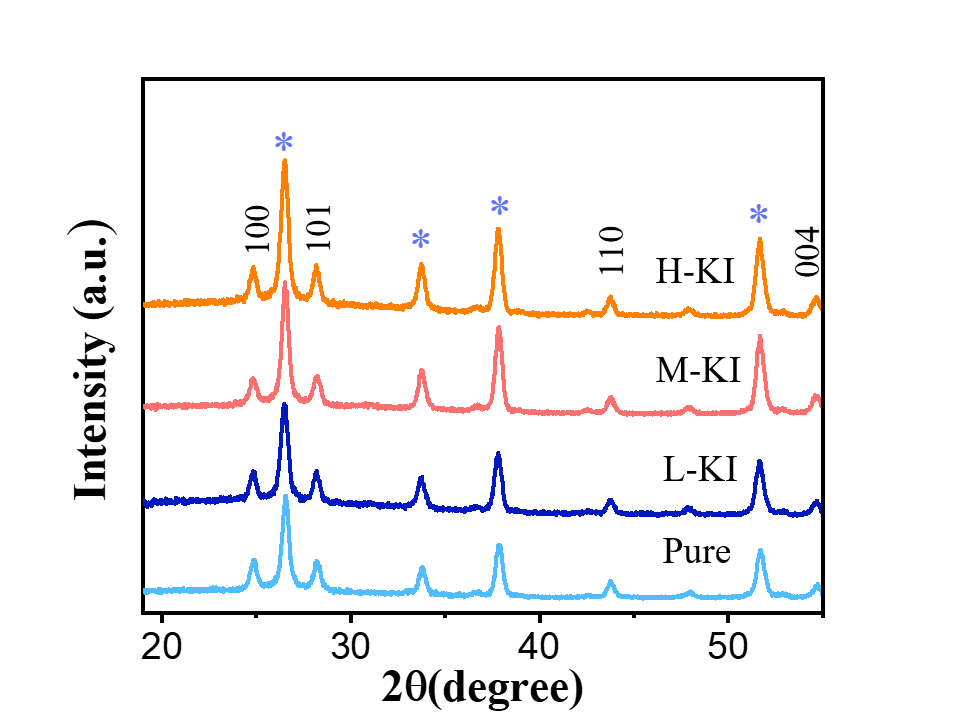
**

**Figure S1**. CdS-XRD for diff KI concentrations


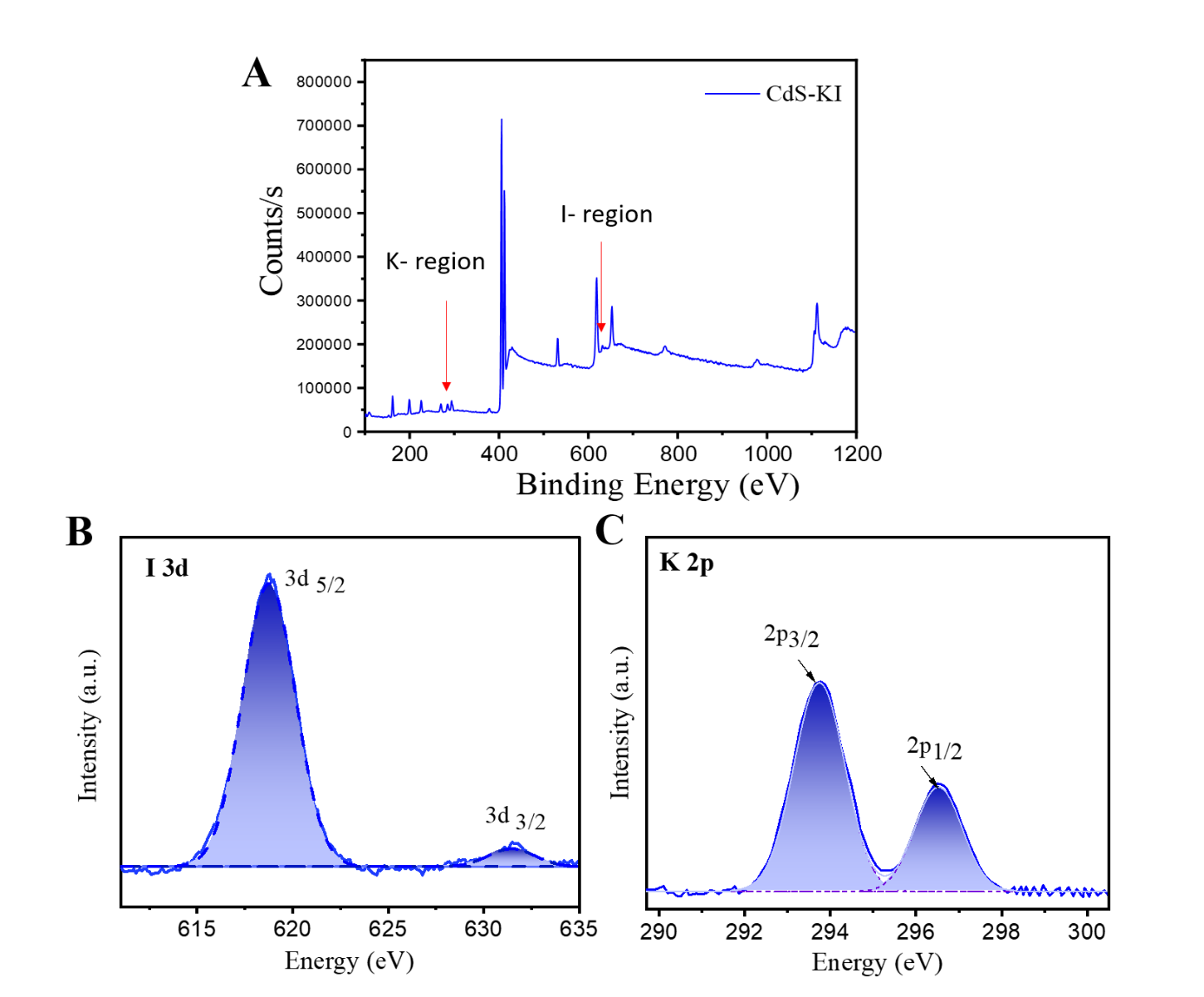


**Figure S2**. (A) Full XPS spectrum for of CdS-KI film without annealing (B) highlighted I 3d-region (C) highlighted K 2p region.


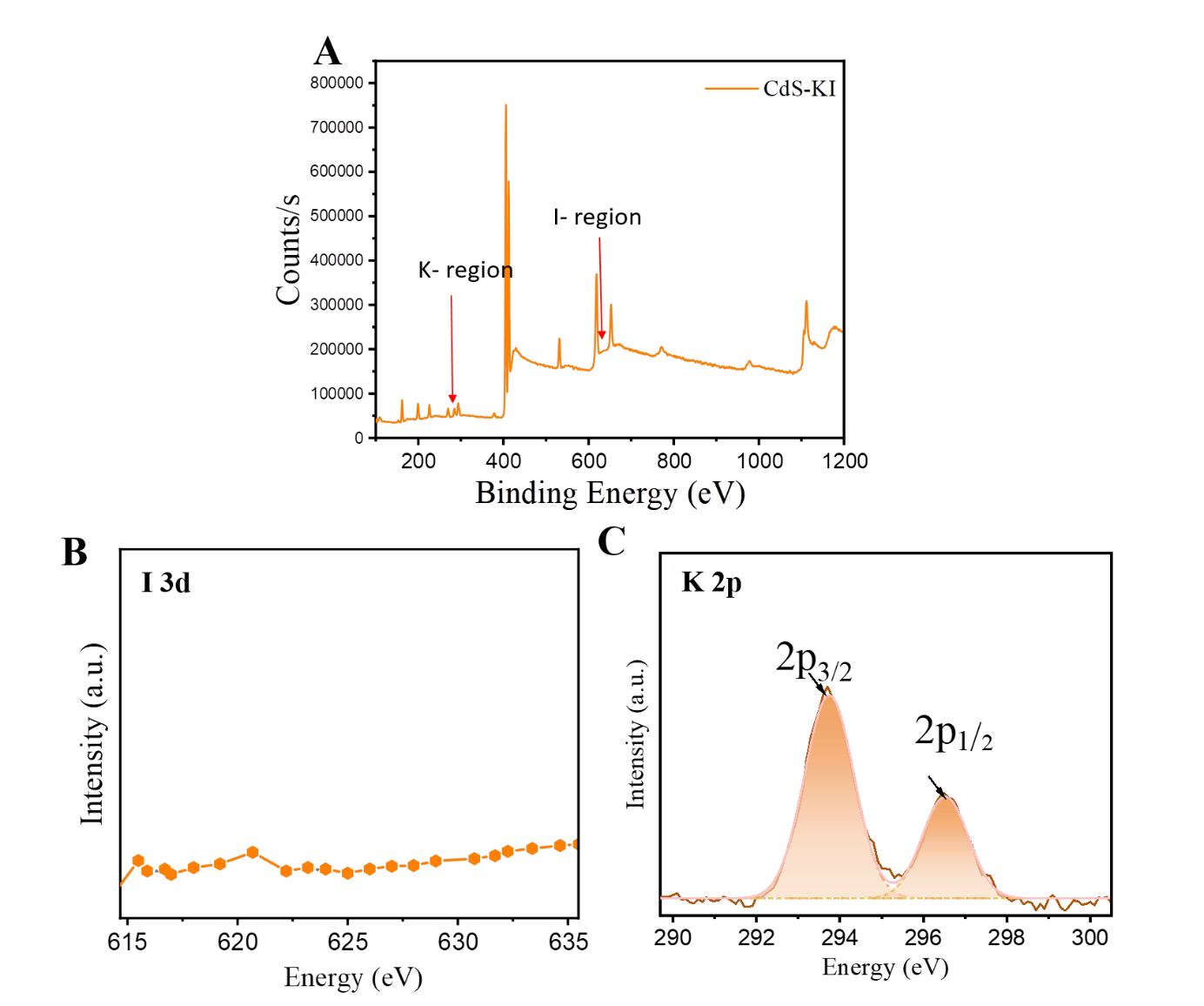


**Figure S3**. (A) Full XPS spectrum for of CdS-KI film after annealing (B) highlighted I 3d-region (C) highlighted K 2p region.


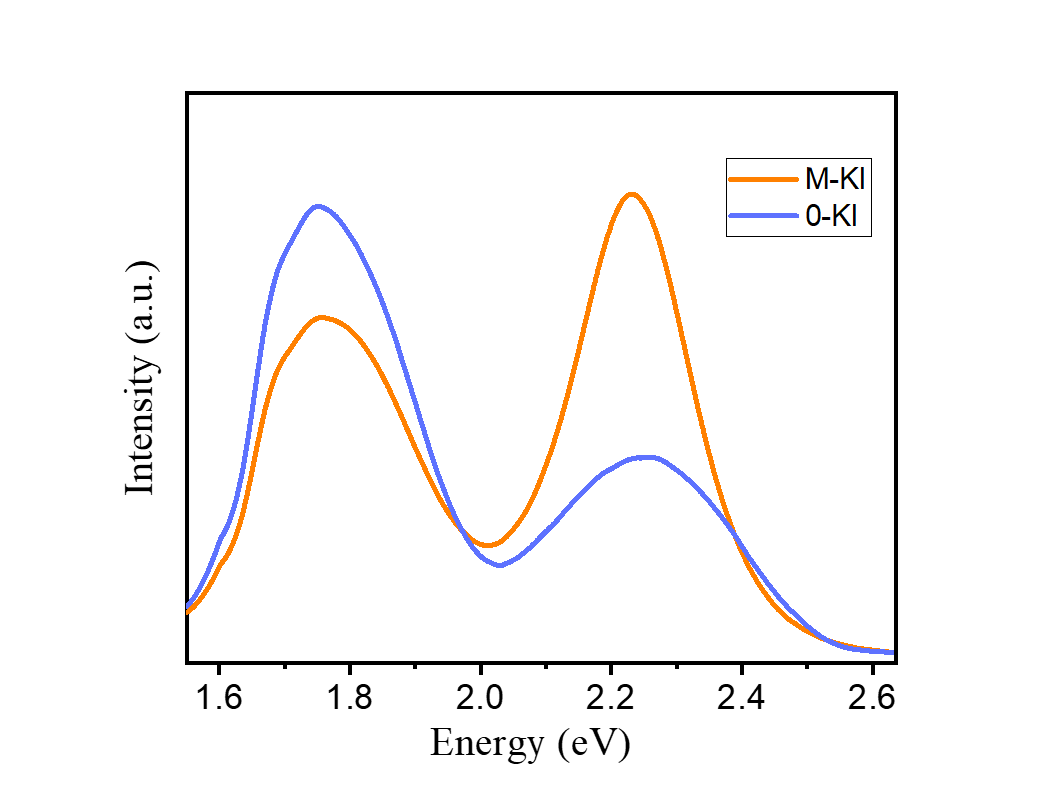


**Figure S4**. PL spectra for pure CdS (0-KI) and optimized KI treated CdS (M-KI)


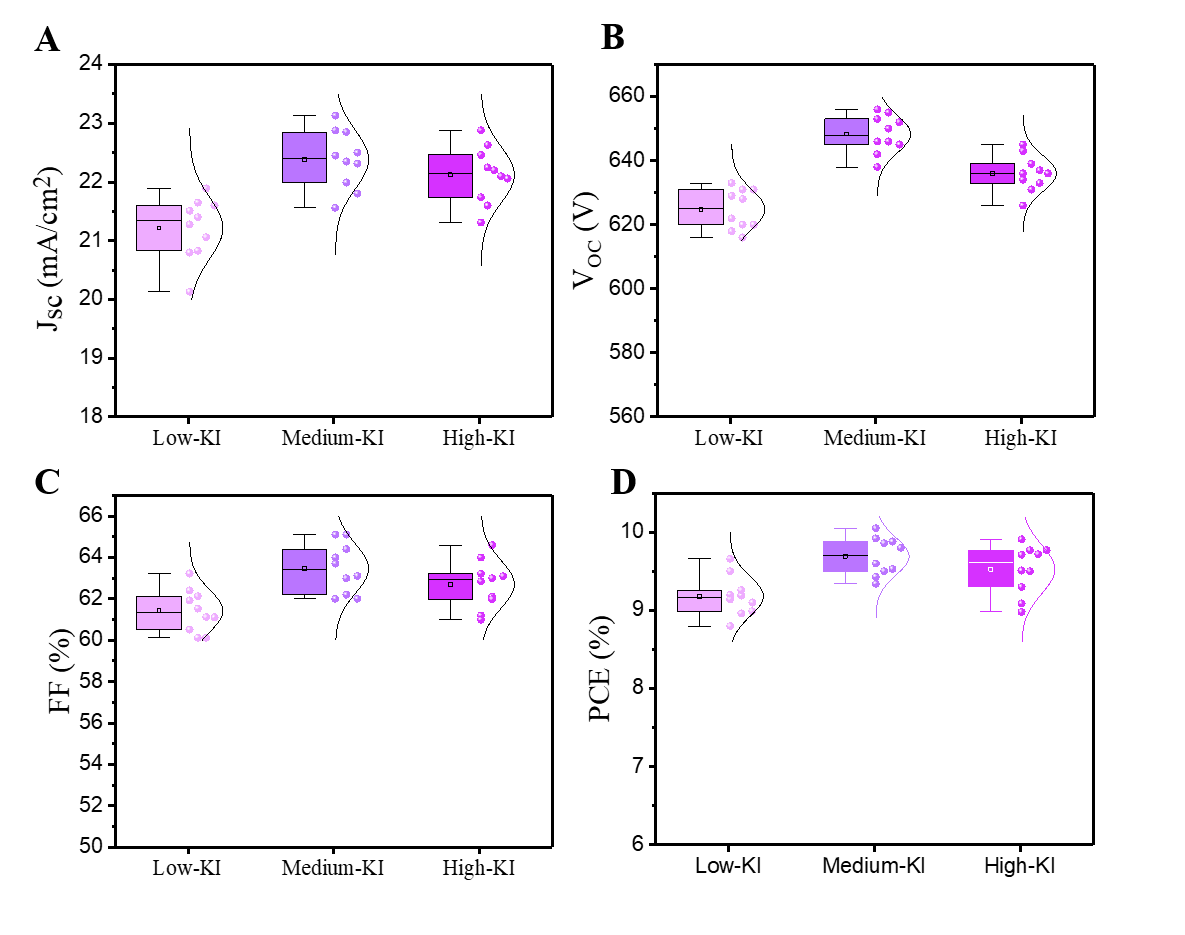


**Figure S5**. Solar cell performance evolution of Sb_2_(S,Se)_3_ film for different KI. A total of 10 devices were analyzed for each measurement


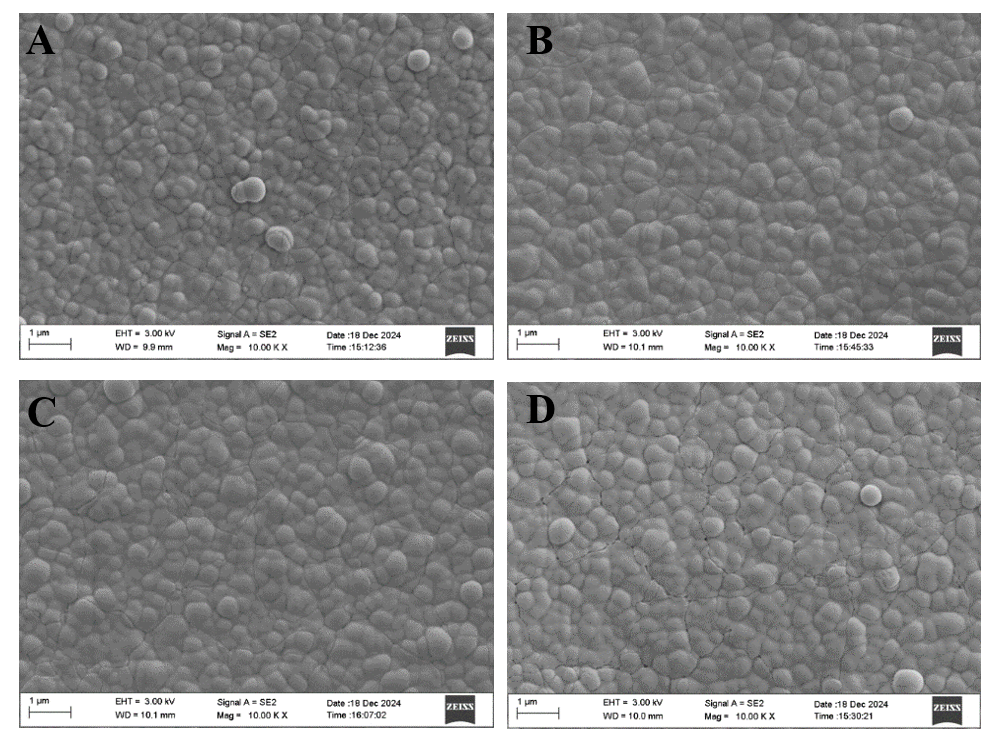


**Figure S6**. SEM imag for (A) pure Sb_2_(S,Se)_3_ film , and urface morphology evolution of Sb_2_(S,Se)_3_ film for different KI concentration increasing from (B-D), i.e., L-KI, M-KI H-KI, respectively).


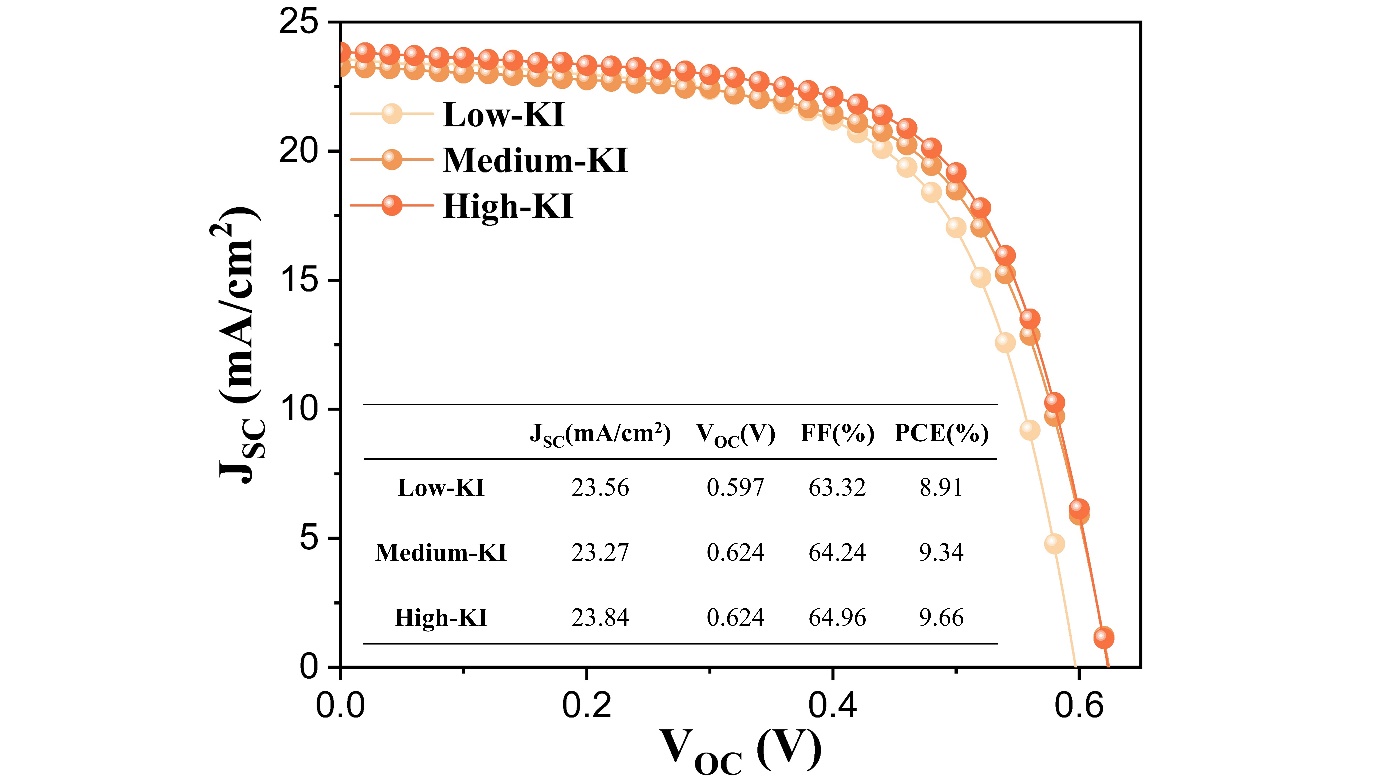


**Figure S7.** Current–Voltage curves for top performing devices with post-deposited KI solution with different concentrations.


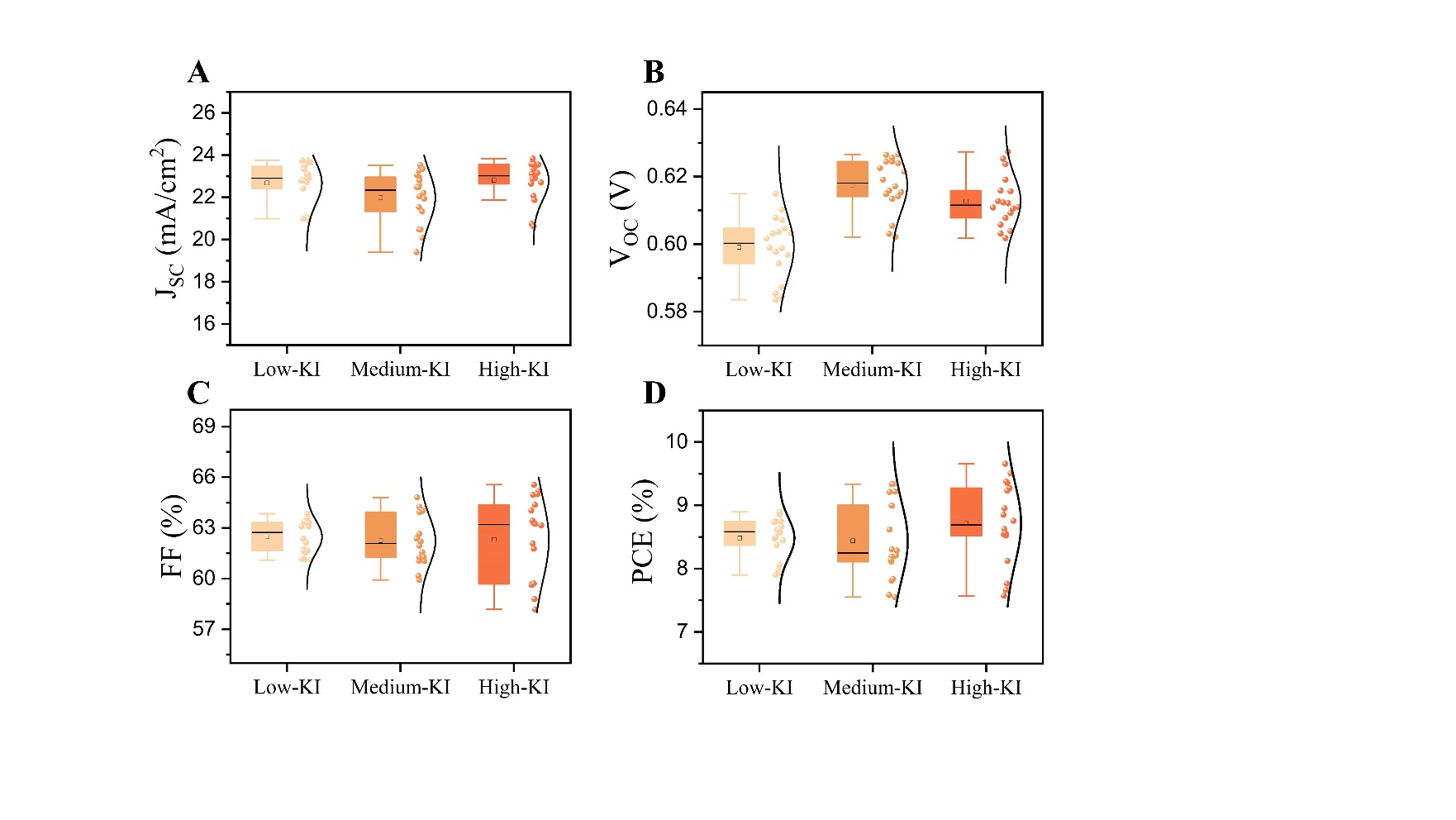


**Figure S8.** Statistical distribution of photovoltaic parameters for post-deposited KI solution with different concentrations: (A) Jsc, (B) Voc, (C) FF, and (D) PCE. A total of 18 devices were analyzed for each measurement.


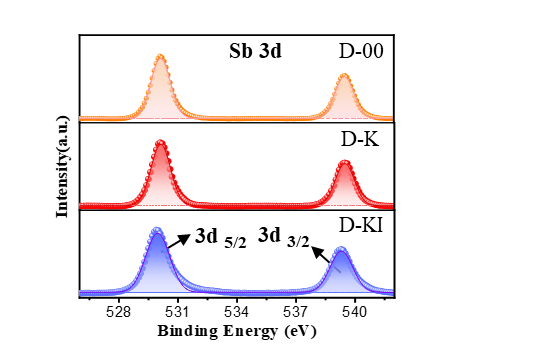


**Figure S9**. XPS spectra for Sb 3d peaks of the absorber films


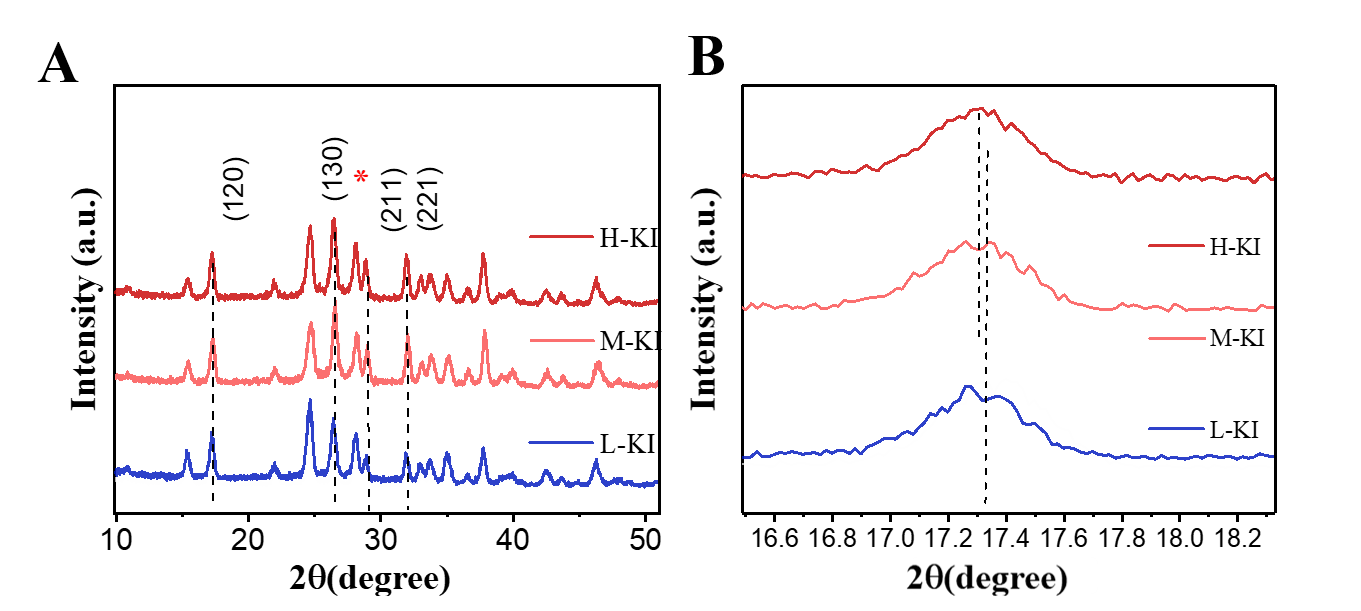


**Figure 10.** (A) X-ray diffraction plot of the absorber film for different KI concentration (B) highlighted (130) peaks


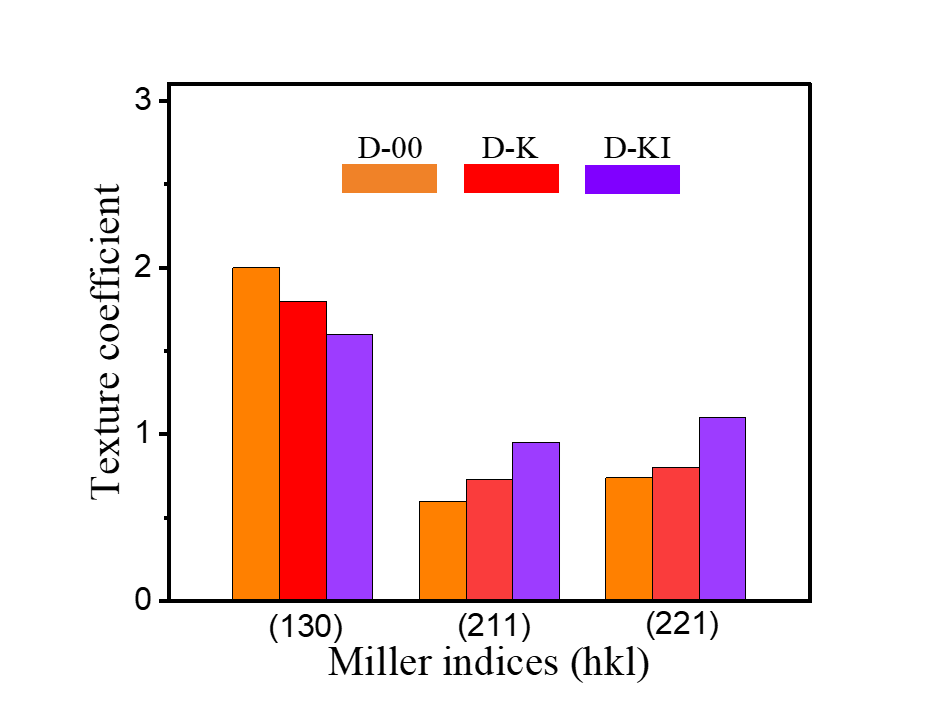


**Figure S11**. Taxture coeeficient analysis of X-ray diffraction plot for D-00, D-K and D-KI films.


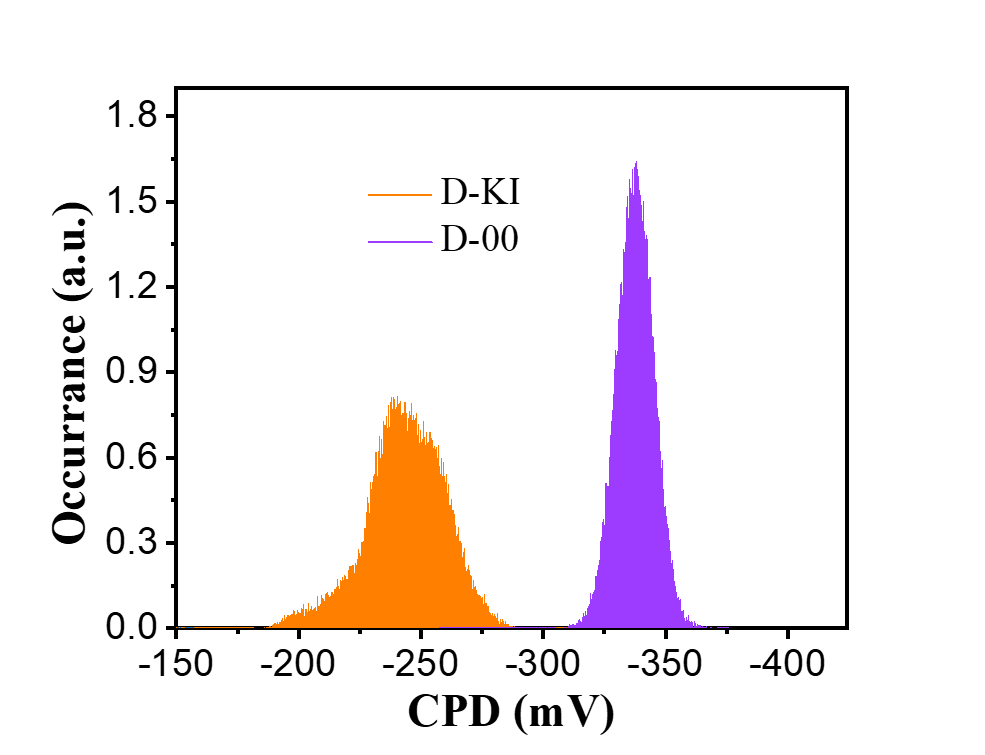


**Figure S12**. Surface potential of D-00 and D-KI device


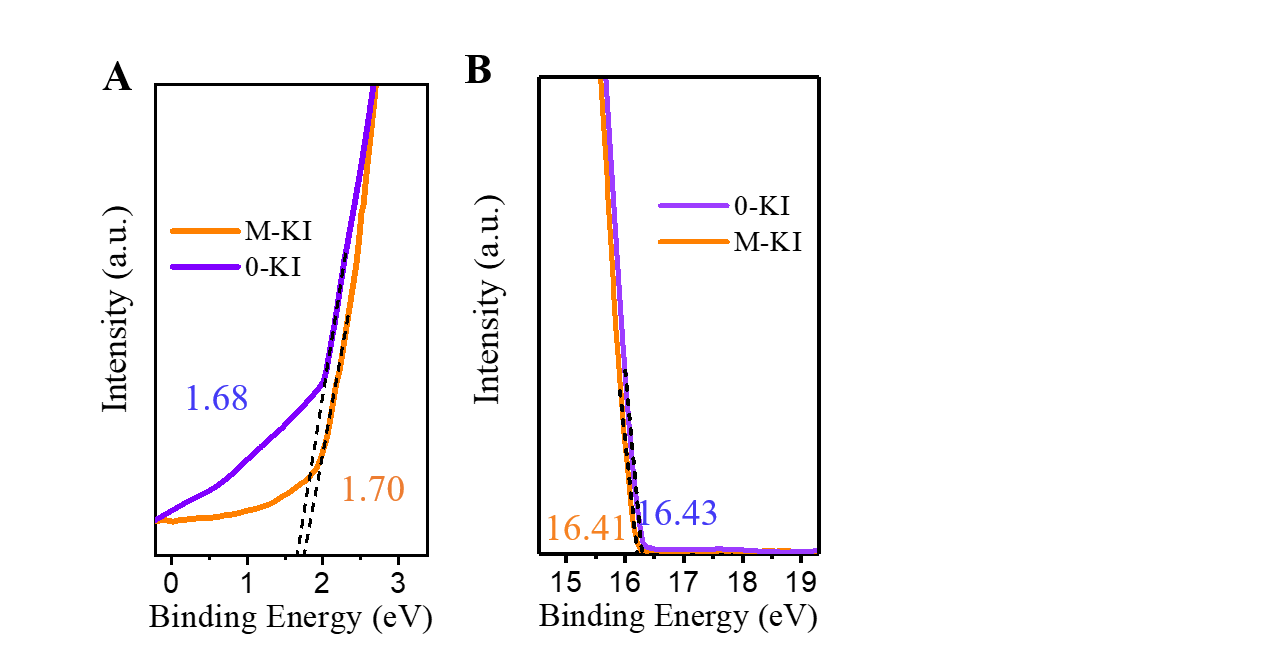


**S13**: UPS curves for pure CdS (0-KI) and optimized Ki treated (M-KI) film


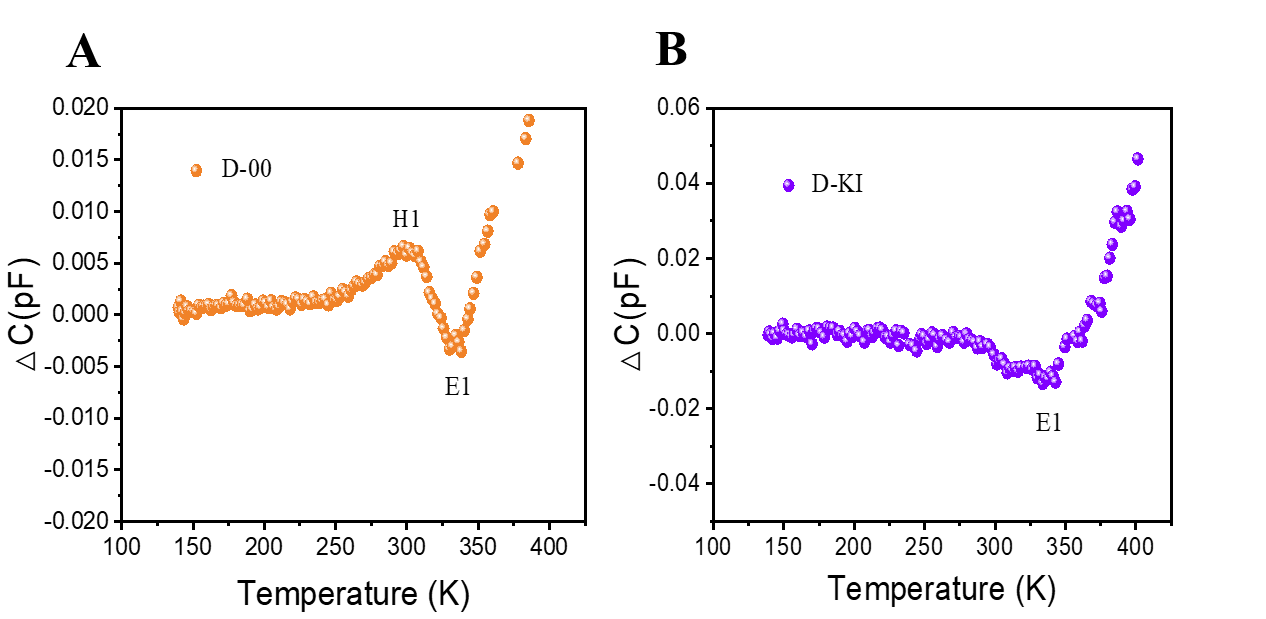


**S14**: DLTS signals for the device without and with KI incorporations


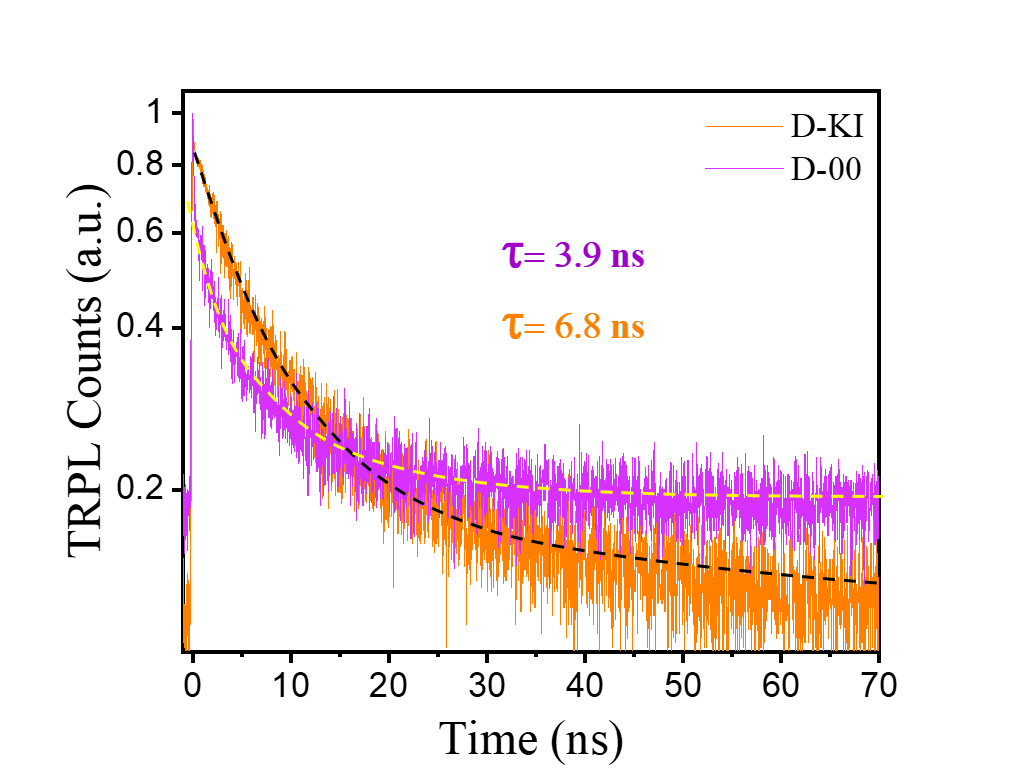


**S15**: TRPL spectra for pure (D-00) and KI-treated (D-KI) films.

**Note 1:** Analysis of dark J–V curves of KI-treated and untreated devices

As the exciton separation is implemented by junction interface, thus the junction ideality factor (A) plays the key role for device performance. Firstly, we analyzed A to investigate the improvement mechanism. The abrupt junction *J–V* equation can be expressed as [1,2]

$J=J_{0}exp\left[ \frac{q}{AkT}\left( V-RJ \right) \right]+GV-J_{L}$ (1)

In the above equation, J_0_, G, and JL are reverse saturation current of the device under dark conditions, conductance under shunt condition, and current density of the device, respectively. We further took differential of equation (1) with respect to J, which is

$\frac{dV}{dV}=R+\frac{AkT}{q} {(J+J_{L})}^{-1}$ (2)

Thus from the flat region of dJ/dV curve, the G value can be extracted. Furthermore, dV/dJ over (J+J_SC_)^-1^ curves linear fit can reveal series resistance (R_S_) and while the slop of the plot (AkT/q) can be used to calculate ideality factor (A). By plotting log of J+J_SC_+GV over V-RJ, the J_0_ value can be calculated. It is to be noted that, q is elementary charge, T is the absolute temperature, and k is Boltzmann constant.

**Note 2:** Extraction of energy lavel data from UPS analysis

Fermi energy (E_F_) of KI treated CdS film was derived by subtracting the intercept at a binding energy of 16.41 eV from the excitation source of 21.2 eV, which gives the E_f_ value (4.79 eV) [3]. The linear fitting value of UPS spectrum tail (1.70 eV) is the distance between Fermi energy and valance band maximum (VBM). Thus the E_V_ is -6.49 eV. Finally adding the bandgap of Ki-CdS to E_V_ give us the value for conduction band (E_C_), which is 4.02 eV. By same way we calculated the energy levels for pure CdS, and those were very similar to reported values for CdS in our previous works. It is important to mention that the gap between E_C_ and E_f_ for KI treated CdS was a slightly shorted, thus ensuring better conductivity type.

Similar calculation was conducted to find the energy level positions for Sb_2_(S,Se)_3_ film with and without KI treatment, which are presented in the main text to draw energy level diagrams. The lover binding energy of the UPS curves for D-00 and D-KI films were 1.01 eV and 0.91 eV, while the intercept at high binding energy were 16.80 eV and 16.46 eV, respectively. It is important to highlight here that the fluctuation in the energy levels are aligned with the finding in other supporting characterizations such KPFM. The negative signs with binding energy values are omitted to avoid confusion in this note.

**Note 3:** Extraction of decay time from TRPL

Time-resolved photoluminescence (TRPL) analysis was conducted on both the films using 470 nm excitation light. TRPL is generally used for investigating the lifetime of photogenerated carriers [4]. The spectra displayed in Figure S12 were fitted using a biexponential function to obtain the fitting parameters. Where τ_1_ is the shorter decay component and τ_2_ is the long decay component, and A_1_ and A_2_ are their corresponding weighting factors. The average lifetime was calculated according to the following equation. While the detailed fitting parameters are provided as well in table below.

$\tau=(A1\tau1+A2\tau2)/(A1+A2)$ (3)

**Table S1**: Detailed fitting parameters for TRPL

| Samples | τ_1_(ns) | τ_1_(ns) | A1 | A2 | τ (ns, avg) |
| --- | --- | --- | --- | --- | --- |
| D-00 | 3.0 | 12.0 | 0.6 | 0.4 | 6.61 |
| D-KI | 2.5 | 7.0 | 0.7 | 0.3 | 3.85 |

**References**

[1] M. Ishaq, H. Deng, S. J. Yuan, H. Zhang, J. Khan, U. Farooq, H. S.Song, J. Tang, High Open-Circuit Voltage in Full-Inorganic Sb_2_S_3_ Solar Cell via Modified Zn-Doped TiO_2_ Electron Transport Layer. *Sol. RRL* **2018**, 2, 1800144

# [2] L. Wang, M. Luo, S. Qin, X. Liu, J. Chen, B. Yang, M. Leng, D.-J. Xue, Y. Zhou, L. Gao, H. Song, J. Tang, Ambient CdCl_2_ treatment on CdS buffer layer for improved performance of Sb_2_Se_3_ thin film photovoltaics. *Appl. Phys. Lett*. 2015, 107, 143902

[3] H. Deng, S. Yuan, X. Yang, F. Cai, C. Hu, K. Qiao, J. Zhang, J. Tang,H. Song, Z. He, Efficient and stable TiO_2_/Sb_2_S_3_ planar solar cell from absorber crystallization and Se-atmosphere annealing. *Mater. Today Energy* **2017**, 3, 15

[4] Y. Zeng, K. Sun, J. Huang, M. P. Nielsen, F. Ji, C. Sha, S. Yuan, X. Zhang, C. Yan, X. Liu, H. Deng, Y. Lai, J. Seidel, N. Ekins Daukes, F. Liu, H. Song, M. Green, X. Hao, Quasi-Vertically-Orientated Antimony Sulfide Inorganic Thin-Film Solar Cells Achieved by Vapor Transport Deposition. *ACS Appl. Mater. Interfaces* **2020**, 12, 22825
